# Evidence for Infant-directed Speech Preference Is Consistent Across Large-scale, Multi-site Replication and Meta-analysis

**DOI:** 10.1162/opmi_a_00134

**Published:** 2024-04-03

**Authors:** Martin Zettersten, Christopher Cox, Christina Bergmann, Angeline Sin Mei Tsui, Melanie Soderstrom, Julien Mayor, Rebecca A. Lundwall, Molly Lewis, Jessica E. Kosie, Natalia Kartushina, Riccardo Fusaroli, Michael C. Frank, Krista Byers-Heinlein, Alexis K. Black, Maya B. Mathur

**Affiliations:** Department of Psychology, Princeton University; Department of Linguistics, Cognitive Science and Semiotics, School of Communication and Culture, Aarhus University; Interacting Minds Center, School of Culture and Society, Aarhus University; Osnabrück University of Applied Sciences; Department of Psychology, Stanford University; Department of Psychology, University of Manitoba; Department of Linguistics and Scandinavian Studies, University of Oslo; Psychology Department and Neuroscience Center, Brigham Young University; Department of Psychology/Social and Decision Sciences, Carnegie Mellon University; Department of Psychology, Concordia University; School of Audiology and Speech Sciences, University of British Columbia; Quantitative Sciences Unit, Stanford University

**Keywords:** infant-directed speech, meta-analysis, mega-analysis, multi-lab replication, looking time preference

## Abstract

There is substantial evidence that infants prefer infant-directed speech (IDS) to adult-directed speech (ADS). The strongest evidence for this claim has come from two large-scale investigations: i) a community-augmented meta-analysis of published behavioral studies and ii) a large-scale multi-lab replication study. In this paper, we aim to improve our understanding of the IDS preference and its boundary conditions by combining and comparing these two data sources across key population and design characteristics of the underlying studies. Our analyses reveal that both the meta-analysis and multi-lab replication show moderate effect sizes (*d* ≈ 0.35 for each estimate) and that both of these effects persist when relevant study-level moderators are added to the models (i.e., experimental methods, infant ages, and native languages). However, while the overall effect size estimates were similar, the two sources diverged in the effects of key moderators: both infant age and experimental method predicted IDS preference in the multi-lab replication study, but showed no effect in the meta-analysis. These results demonstrate that the IDS preference generalizes across a variety of experimental conditions and sampling characteristics, while simultaneously identifying key differences in the empirical picture offered by each source individually and pinpointing areas where substantial uncertainty remains about the influence of theoretically central moderators on IDS preference. Overall, our results show how meta-analyses and multi-lab replications can be used in tandem to understand the robustness and generalizability of developmental phenomena.

## INTRODUCTION

Across many cultures, adults adjust the way they speak with infants compared to how they speak with other adults (Cox et al., 2023; Fernald et al., 1989; Hilton et al., 2022). This type of speech addressed to infants (infant-directed speech, or IDS) has unique acoustic and linguistic characteristics compared with adult-directed speech (ADS): for example, IDS tends to be produced with a slower articulation rate, a greater degree of pitch variability, and acoustically exaggerated vowels (Hilton et al., 2022; Kalashnikova & Burnham, 2018; Singh et al., 2002; Stern et al., 1983). Decades of research have investigated infants’ responsiveness to this distinctive style of speech, finding that infants prefer IDS over ADS from a young age (Cooper & Aslin, 1990; Fernald & Kuhl, 1987; Pegg et al., 1992; Werker & McLeod, 1989) and that this preference persists even when the speech is filtered to contain only prosodic information (Fernald & Kuhl, 1987) or when presented in a foreign language (The ManyBabies Consortium, 2020). IDS has been argued to play an important role in supporting early language and cognitive development, with the speech style initially serving primarily to draw infants’ attention, modulate their temperament and express affect, and later serving more specific linguistic and non-linguistic purposes (Cox et al., 2023; Csibra & Gergely, 2009; Eaves et al., 2016; Fernald et al., 1989; Hartman et al., 2017; Peter et al., 2016; Snow & Ferguson, 1977; Soderstrom, 2007).

Given its centrality in theories of language and cognitive development, how robust is the evidence for infants’ IDS preference? A substantial body of research on the IDS preference has culminated in both i) a community-augmented meta-analysis (MA) of published behavioral studies (Anderson et al., 2021; Dunst et al., 2012) and ii) a multi-lab replication (MLR) study (The ManyBabies Consortium, 2020). How can we compare and synthesize the findings from these two different types of data sources? The aim of this paper was to improve our understanding of the relationship between MA and MLR evidence and to determine the generalizability and boundary conditions of the IDS effect across theoretically relevant dimensions.

The first source of evidence we considered was a community-augmented MA of the IDS preference—a meta-analysis with openly accessible data that can be dynamically updated through new contributions from the research community (Tsuji et al., 2014). This MA was developed based on a previously published MA that analyzed 16 papers with a total of 51 effect sizes published between 1983 and 2011 (Dunst et al., 2012). In the published meta-analysis, infants generally preferred to listen to IDS over ADS speech stimuli (Cohen’s *d* = 0.67, 95% CI [0.57, 0.76]). This report also documented variability across several moderators, including that (i) older infants exhibited a stronger preference to attend to IDS over ADS than younger infants and (ii) that characteristics of the methodological design and stimuli systematically affected IDS preference (e.g., effects were stronger if speakers were unfamiliar to infants). The original Dunst et al. MA was subsequently revised and augmented (see Methods for details) by the MetaLab community of infant researchers, resulting in a MA encompassing 30 papers published between 1985 and 2020 that contributed a total of 112 effect sizes (https://metalab.stanford.edu; Anderson et al., 2021; Bergmann et al., 2018). Notably, this community-augmented meta-analysis resulted in a substantially smaller effect size estimate (*d* = 0.35, 95% CI [0.22, 0.47]).

Our second source of evidence was a MLR of IDS preference, in which 69 laboratories on four continents (Asia, Australia, Europe, North America) collected data from over 2700 infants aged between 3 and 15 months (The ManyBabies Consortium, 2020). The general aim of ManyBabies 1 was to replicate the main phenomenon of IDS preference among infants while assessing the impact of several theoretically meaningful variables, including infant age, language experience and testing methods. IDS preference was measured by analyzing infants’ behavioural visual responses to IDS and ADS speech stimuli using three different methods that were self-selected by each participating lab: central fixation, the head-turn preference procedure (HPP) and eye-tracking. The sets of ADS and IDS stimuli were held constant across laboratories and were created by recording a small number of North American mothers in a semi-naturalistic speech elicitation task. The results from the MLR indicated i) that infants generally prefer to listen to IDS over ADS speech stimuli (overall Cohen’s *d* = 0.35, 95% CI [0.29, 0.42]), ii) that the preference for IDS over ADS was strongest in the oldest age range tested, iii) that infants learning North American English (i.e., whose native language matched that of the test stimuli) showed stronger effects than those learning languages other than North American English, and iv) that the HPP elicited stronger effects than both central fixation and eye-tracking.

On the surface, the evidence for the IDS preference appears broadly consistent across the MLR and the community-augmented MA: both sources show small-to-moderate positive effect sizes. However, these studies take fundamentally different approaches to deriving their overall estimates, with distinctive strengths and weaknesses. MAs have traditionally been considered a gold standard form of evidence, by offering a bird’s-eye view of the generalizability of a phenomenon—as well as the heterogeneity of effects—across a variety of designs and populations. However, they have also been criticized on several grounds (Corker, 2022; Lakens et al., 2016; Siddaway et al., 2019; Stanley, 2001). One major concern is that MAs are subject to publication bias (Sterne et al., 2001). MAs are often limited to the available (published and grey) literature, and a small set of unpublished studies individual researchers are willing and able to dredge from the file drawer. This limitation may bias estimates, as positive results are typically over-represented in the published literature (Masicampo & Lalande, 2012; Mathur & VanderWeele, 2021a; McShane & Gal, 2017; Sterne et al., 2001). A second concern is that heterogeneity in the studies included in a meta-analysis can threaten to complicate practical interpretation when taken to an extreme, i.e., meta-analyses may be comparing “apples to oranges” (Eysenck, 1978; Simonsohn et al., 2022). While there are statistical approaches that attempt to correct for publication bias and measure and account for heterogeneity, major concerns about the validity of MA results—even when corrected—remain.

In part due to the limitations of MAs, many researchers have begun to consider MLRs the new gold standard. In such designs, multiple labs conduct replications of original studies by implementing a common experimental protocol to test a research question across sites (e.g., Ebersole et al., 2016, 2020; Jones et al., 2021; Klein et al., 2014, 2018, 2019; The ManyBabies Consortium, 2020). Like MAs, MLRs (such as ManyBabies 1) can achieve larger aggregated sample sizes than are typical in single-lab studies, but the similarity in implementation across labs may offer greater comparability within the dataset. Moreover, MLRs do not suffer from concerns about publication bias, because the results from all labs are reported transparently regardless of outcome. On the other hand, more uniformity in experiment implementation may lead to effect size estimates that are less robust to methodological and analytical differences; that is, the measured effect size may reflect the particular methodological and analytic choices of the study. Therefore, more narrowly defined experimental parameters may limit the degree to which MLRs can speak to the generalizability and boundary conditions of a phenomenon (Visser et al., 2022; Yarkoni, 2020).

While both MAs and MLRs individually represent valuable methods for estimating an effect of interest, consulting either a MA or MLR in isolation likely provides an incomplete picture of theoretically important phenomena. Furthermore, past work comparing MAs and MLRs suggests that the results obtained from these two approaches often do not agree. In a study that systematically compared 15 pairs of published MAs and MLRs within the field of psychology, Kvarven et al. (2020) found significant differences in mean effect sizes for 12 of the pairs, with MA effect sizes on average three times the size of those obtained via MLRs. What drives these differences remains unclear. For example, in a reanalysis of the same data, Lewis et al. (2022) concluded that these discrepancies could not be fully accounted for by publication bias. An alternative explanation appeals to potential heterogeneity in the MA (Lewis et al., 2022). If there is true heterogeneity in the studies summarized in the MA, this could create a false impression of inconsistent results between the MA and the MLR, despite the MLR estimate falling within a reasonable subdistribution of effects in the MA. Given the limitations of MAs and MLRs considered alone—and resulting divergences in the conclusions derived from each method—, a promising approach to understanding a key phenomenon of interest is to combine and synthesize evidence from both sources. This strategy seems particularly useful given how the benefits of each approach may counteract the limitations of the other. MLRs can provide estimates that do not suffer from publication bias, whereas MAs can typically offer estimates across a wider variety of experimental design choices than MLRs.

In the current paper, we investigate the overall magnitude, generalizability, and boundary conditions of the IDS preference effect by integrating and comparing experiment-level data from both the MA and MLR. Unlike past comparisons focusing on the overall effect size of MAs and MLRs (e.g., Kvarven et al., 2020), we explicitly model data from the individual studies included in the MA and individual experiments contributing to the MLR. We simultaneously code key features of each experiment to investigate whether heterogeneity in effects across moderating variables thought to substantially impact IDS preference can explain any discrepancies between the MA and MLR. We take a meta-regression approach, estimating the magnitude of IDS preference aggregating across the two data sources with and without theoretically-motivated moderator-level variables. Together, these analyses increase our overall understanding of IDS preference while also providing a detailed case study of the relationship between MA and MLR. We focus on three main questions:

1. Do the MA and MLR provide comparable estimates of infant preference for IDS?
2. Does accounting for study-level moderators and publication bias affect the comparison of the estimates across the two approaches?
3. Are there differences between the MA and the MLR in how study-level moderators predict IDS preference?

The first two questions followed a preregistered analytic approach, while the third question was investigated in additional exploratory analyses. The preregistered analyses were designed to be conducted using the original Dunst et al. (2012) meta-analysis as the main MA source. However, after the preregistered plan was finalized, two key events occurred: (a) we uncovered substantial issues with coding decisions in the original meta-analysis that required revision and (b) the original meta-analysis was augmented via systematic search to include almost twice the number of studies (see Methods and Supplementary Materials). In order to test our primary research questions with the most extensive and accurate evidence source possible, we therefore opted to deviate from the preregistration and execute our preregistered analytic plan using the community-augmented MA as our primary meta-analytic data source.^1^

## METHODS

All confirmatory analyses were preregistered prior to data analysis at https://osf.io/scg9z. The Supplementary Materials provide further details on the preregistration framework (Section 1.1) and deviations from our preregistered plan (Section 1.2), and contextualizes the updates to datasets (Section 6).

### Meta-analysis

#### The Original Dunst et al. (2012) Meta-analysis.

The MA by Dunst et al. (2012) reports study-level effect sizes in Appendix C of the original study and moderator variables in Appendices A and B. We digitized these variables, and an independent team checked and corrected the resulting spreadsheet to fully reflect the published meta-analysis. We additionally computed effect size variances using standard formulae based on reported standardized mean difference (SMD) and sample sizes. To supplement the MA with moderators that were relevant for the research questions in this study but not reported on in the MA (Dunst et al., 2012), it was necessary to re-examine the papers reporting on the original experiments. This process led to a number of additional moderating variables that included further detail about (1) whether the test language was native for infant participants, non-native, or an artificial language; (2) whether the main question of the study was focused on IDS preference; (3) variation in experimental methods (e.g., whether test trials were infant-controlled or had a fixed duration); and (4) variation in participant exclusions and exclusion criteria (e.g., what number of test trials were required for infant inclusion).

#### Revisions to the Dunst et al. Meta-analysis.

When coding for additional moderators for the studies included in the original MA (Dunst et al., 2012), we encountered substantial issues, such as incorrectly reported effect sizes and inappropriate inclusion and exclusion of experimental conditions (as discussed further in Section 2.1 of the Supplementary Materials). The original MA never underwent a formal peer review process, which could have caught some of these errors; however, even published and reviewed MAs are not exempt from replicability and reproducibility issues (Maassen et al., 2020; Nuijten et al., 2016).

#### The Community-augmented Meta-analysis.

The revised Dunst et al. meta-analysis was subsequently augmented based on new literature searches conducted in 2017 and 2019, resulting in an updated, community-augmented database of studies on infants’ IDS preference in MetaLab (https://metalab.stanford.edu/; Tsuji et al., 2014). Further details about the augmentation process are provided in Section 2.2 of the Supplementary Materials. The community-augmented MA comprised *k* = 30 studies contributing a total of *m* = 112 estimates, which included a median of *n* = 16.50 participants.

To provide the most comprehensive, up-to-date point of comparison between the MLR and the MA, we focus our preregistered analyses on the updated, community-augmented MA that includes revisions to the issues identified in the original meta-analysis. All analyses using the original dataset (i.e., Dunst et al., 2012) and a revised version containing only studies included in the original MA (i.e., correcting errors or other issues in the coding of papers from the original MA, but not updating the dataset to include additional studies)—as well as a discussion of differences with the main conclusions presented here—can be found in the Supplementary Materials (Section 5 and Section 6).

### Multi-Lab Replication: ManyBabies 1

A total of *k* = 62 labs contributed a total of *m* = 102 estimates to the dataset, because single labs could contribute data in multiple age groups. This dataset is identical to the data in the original analyses (The ManyBabies Consortium, 2020), which excluded infants who did not provide at least one trial per condition (IDS and ADS in paired trials) and labs providing estimates from less than ten infants. Note that slightly fewer labs were included in this analysis in The ManyBabies Consortium (2020) compared to the overall number of labs contributing to the project (*N* = 67) because of stricter inclusion criteria (infants were required to contribute paired IDS and ADS trials). The data were downloaded from the public GitHub repository (https://github.com/manybabies/mb1-analysis-public) of the MLR. Effect sizes were computed, both here and in the original paper, as standardized mean differences (SMD) based on the average looking time difference in IDS and ADS trials divided by the pooled standard deviation of looking time on the level of study (i.e., an age group within a lab); variance was computed accordingly. Post hoc, we added all moderators that were not part of the original dataset, such as speaker identity (e.g., unfamiliar female), to align this dataset with the MA (see Table 1). The estimates in this dataset are based on a median of *n* = 16 participants per age group (ranging from 10 to 46). For further details on the MLR, including participant sampling and exclusion criteria, see sections 3.1 and 3.2 of the Supplementary Materials.

**Table 1.** The distribution of moderators in the community-augmented meta-analysis (MA) and multi-lab replication (MLR).

		MA	MLR
Number of effect sizes		112	102
Infant age (months; centered)	Mean (SD)	0.00 (5.76)	1.22 (3.03)
Test language			
	Native	103 (92.0%)	46 (45.1%)
	Non-Native	6 (5.4%)	56 (54.9%)
	Artificial	3 (2.7%)	0 (0%)
Native language			
	Cantonese	4 (3.6%)	0 (0%)
	Dutch	0 (0%)	5 (4.9%)
	English	103 (92.0%)	62 (60.8%)
	French	0 (0%)	6 (5.9%)
	German	0 (0%)	14 (13.7%)
	Hungarian	0 (0%)	2 (2.0%)
	Italian	0 (0%)	1 (1.0%)
	Japanese	5 (4.5%)	4 (3.9%)
	Korean	0 (0%)	3 (2.9%)
	Norwegian	0 (0%)	1 (1.0%)
	Spanish	0 (0%)	2 (2.0%)
	Swiss German	0 (0%)	1 (1.0%)
	Turkish	0 (0%)	1 (1.0%)
Experimental method			
	Central Fixation	67 (59.8%)	68 (66.7%)
	HPP	39 (34.8%)	34 (33.3%)
	Other	6 (5.4%)	0 (0%)
Speech type			
	Simulated	75 (67.0%)	0 (0%)
	Naturalistic	30 (26.8%)	102 (100%)
	Filtered or Synthesized	7 (6.3%)	0 (0%)
Speaker familiarity (own mother)			
	No	109 (97.3%)	102 (100%)
	Yes	3 (2.7%)	0 (0%)
Mode of presentation			
	Audio	93 (83.0%)	102 (100%)
	Video	19 (17.0%)	0 (0%)
Dependent measure			
	Preference	104 (92.9%)	102 (100%)
	Affect	8 (7.1%)	0 (0%)
Main question: IDS preference			
	Yes	88 (78.6%)	102 (100%)
	No	24 (21.4%)	0 (0%)

### Hypothesized Estimate-level Moderators

**Figure 1.**
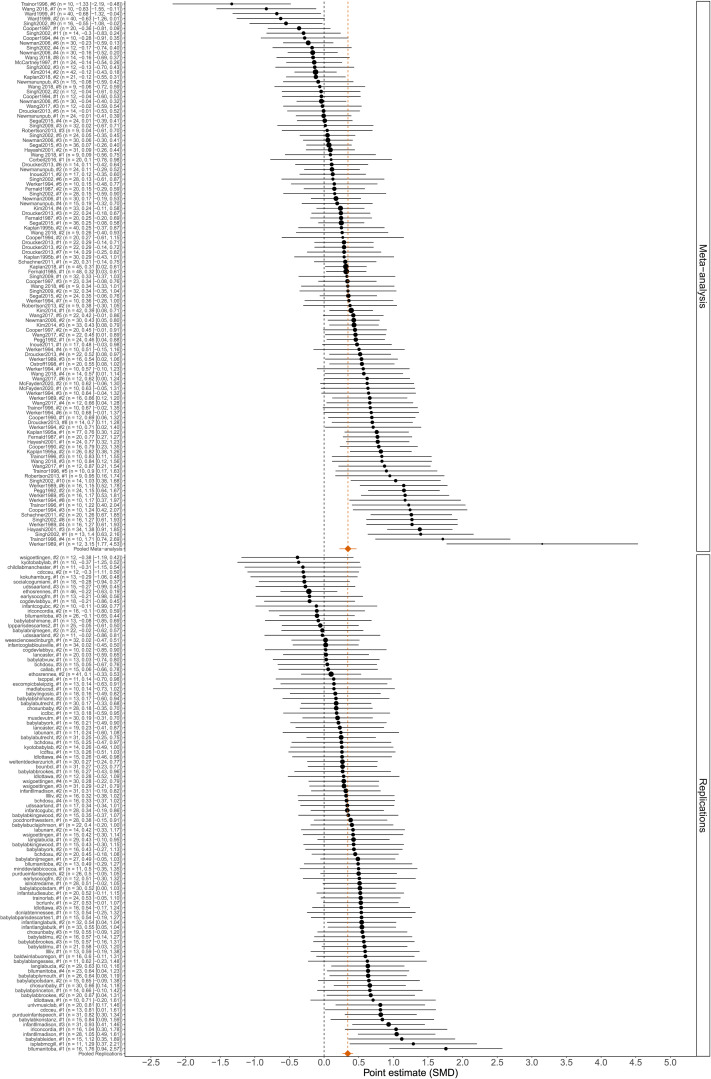
Forest plot of studies’ point estimates and 95% confidence intervals in the MA (top panel) and MLR (bottom panel). Orange diamonds: pooled estimates within each source. Dashed vertical line: null.

In our primary analyses, we investigated eight hypothesized estimate-level moderators of the IDS preference effect, which we coded in both sources (i.e., the MA and the MLR datasets; for an overview, see Table 1; for details, see Section 4.1 of the Supplementary Materials). These comprised one characteristic of the study population (average participant age [in months, mean-centered]), four characteristics of the stimuli (test language, speech type, speaker familiarity, and mode of presentation), two methodological characteristics (experimental method and dependent measure), and an overall estimate characteristic (study goal, i.e., whether infants’ preference for infant- over adult-directed speech was the main research question of a paper). One additional moderator we considered was infants’ native language. However, infants’ native language was heavily skewed towards North American English and is confounded with whether stimuli were presented in infants’ own native language, as any non-native stimuli were North American English across both the MA and the MLR. We thus use this factor only for exploratory analyses but mention it here for completeness (cf. also Figure 1 in Section 4.2 of the Supplementary Materials for more information on the distribution of interactions between moderators). In our regression models, we dummy-coded the binary and categorical moderators such that the reference level represented the most common level in the meta-analysis. Similarly, we centered the single continuous moderator, mean age in months, by its mean in the MA.

### Statistical Analyses

#### Evidence Measures.

We used three metrics to characterize evidence strength for IDS preference in each source and to compare evidence between the sources. First, we estimated the average effect size (SMD) in each source. Examining the difference between sources in these average effect sizes is an important first step, but this approach can exaggerate differences between meta-analyses if effects are highly heterogeneous. In such cases, a fairly large difference between means can occur simply as a result of heterogeneity. By the same token, heterogeneity might lead two meta-analytic estimates to appear similar despite important differences in the underlying evidence base (Mathur & VanderWeele, 2019). For this reason, we also estimated other metrics of agreement that more holistically compare the distributions of effects rather than only their means. As a second metric of evidence strength, we estimated the percentage of population effects^2^ in each source that were positive, representing any preference for IDS regardless of magnitude (Mathur & VanderWeele, 2019, 2020a). As a third metric, for a more stringent assessment, we estimated the percentage of population effects in each source representing only effects that were stronger than *SMD* > 0.2 (Mathur & VanderWeele, 2019, 2020a) (in the predicted direction, i.e., showing an IDS preference).

#### Between-source Discrepancies Before and After Accounting for Hypothesized Moderators.

We fit three meta-regression models predicting effect sizes as standardized mean differences (SMD) in R (R Core Team, 2020).^3^: (1) an **unadjusted model** that compared the two sources (MA and MLR) but did not account for other hypothesized moderators, (2) a **moderated model** that additionally included the other hypothesized moderators, and (3) an exploratory **interaction model** that included the two-way interactions between the moderators and the source of the effect sizes.

The first two models estimated the extent to which the MA and MLR results differed when either ignoring estimate-level moderators (the unadjusted model) or when accounting for them (the moderated model). That is, the unadjusted model estimated average effect sizes for each source, the percentage of positive effects, and the percentage of effects stronger than *SMD* = 0.2 when averaging over the distributions of moderators in each source. In contrast, the moderated model estimated these measures for each source when holding constant all moderators to their average values (in the case of continuous variables) or their most common values (in the case of categorical variables) in the meta-analysis, which we used as reference levels. Both models included all *m* = 214 estimates from both data sources. We anticipated that the moderated model (and consequently, the interaction model) would not be statistically estimable if some moderators were relatively highly correlated, so we removed moderators one-by-one in ascending order of scientific relevance until the model was estimable. Three moderators emerged as estimable in the moderated model: infant age, test language, and experimental method (see Supplementary Materials Section 4 for further details).

Finally, we fit an exploratory model including the two-way interactions between source (MA vs. MLR) and the same three estimable moderators in the moderated model (i.e., infant age, test language, and method). We fit this interaction model because each of these three predictors were significantly related to IDS preference in the original ManyBabies analysis (The ManyBabies Consortium, 2020), but did not reach significance in the moderated model. The interaction model thus served to further investigate this discrepancy by estimating the degree to which the effect of each predictor depended on the data source. For this analysis, we simplified the test language predictor (Native vs. Other) and the method variable (HPP vs. Other) into centered, binary variables (as opposed to three-level categorical variables) in order to achieve model convergence. Note that the results from the moderated model above remain unchanged if the moderator variables are simplified in this manner.

#### Publication Bias.

For the MA, we assessed the possible contribution of publication bias to the results and to between-source discrepancies in average effect sizes. First, we assessed publication bias in the MA using selection model methods (Vevea & Hedges, 1995), sensitivity analysis methods (Mathur & VanderWeele, 2020b), and the significance funnel plot (Mathur & VanderWeele, 2020b). These methods assume that the publication process favors “statistically significant” (i.e., *p* < 0.05) and positive results over “nonsignificant” or negative results, an assumption that conforms well to empirical evidence on how publication bias operates in practice (Mathur & VanderWeele, 2021a; Masicampo & Lalande, 2012; McShane & Gal, 2017). We used visual diagnostics to assess the plausibility of these assumptions. “Publication bias” in this context could reflect the aggregation of multiple sources of bias, including, for example, investigators’ selective reporting of experiments or preparation of papers for submission as well as journals’ selective acceptance of papers.

The sensitivity analysis methods do not estimate the actual severity of publication bias, but rather consider how much results might change under varying degrees of hypothetical publication bias. These methods, unlike the selection model, also accommodate the point estimates’ non-independence within articles, do not make distributional assumptions, and do not require a large number of studies (Mathur & VanderWeele, 2020b). Using the sensitivity analysis methods, we estimated the meta-analytic mean under hypothetical worst-case publication bias (i.e., if “statistically significant” positive results were infinitely more likely to be published than “nonsignificant” or negative results). This worst-case estimate arises from meta-analyzing only the observed “nonsignificant” or negative studies and excluding the observed “significant” and positive studies. We also estimated the amount of hypothetical publication bias that would be required to shift the estimate in the MA to match the estimate in the MLR (Mathur & VanderWeele, 2020b). A previous study estimated publication bias to favor affirmative results by a factor of 4.7 on average in a small sample of developmental psychology MAs (Mathur & VanderWeele, 2021a). Consistent with this finding, we conducted a post-hoc analysis estimating the meta-analytic mean assuming the same level of publication bias.

## RESULTS

### Meta-analysis and MLR Results Modeled Separately

The overall effect size in the MA dataset was *SMD* = 0.35 [0.22, 0.47] (*p* < 0.0001), with considerable heterogeneity (estimated standard deviation of population effects $\hat{\tau }$ = 0.31). This effect size was roughly half the size of the effect size for IDS preference reported in the original MA (Cohen’s *d* = 0.67) by Dunst et al. (2012) (cf., Section 5.1 and  5.2 of the Supplementary Materials), indicating a substantial effect of the revisions and extensions performed as part of the community-augmented meta-analysis process (Tsuji et al., 2014). We estimated that the vast majority of the population effects were positive (86% [83%, 90%]) and that most effects were stronger than *SMD* = 0.2 (64% [61%, 73%]; Table 2). Among only the MLR studies, the estimated average effect size was *SMD* = 0.34 [0.27, 0.42]; *p* < 0.0001) and with less estimated heterogeneity ($\hat{\tau }$ = 0.11) compared to the MA (cf., similar results when applying more stringent participant inclusion criteria on the MLR in Section 5.4.6 of the Supplementary Materials). Descriptively, the meta-analytic effect size in the revised MA was therefore virtually identical to that of the MLR when estimating each effect size separately. For the MLR, we estimated that nearly all of the population effects were positive (100% [96%, 100%]) and that a large majority were stronger than *SMD* = 0.2 (89% [76%, 100%]; Table 2). These results are visualised in Figure 1, which shows positive population effects for studies in both sources, but with the MLR exhibiting more concentration around its average effect size estimate than the MA (see also Figure 7 in the Supplementary Materials Section 5.4.3 for a visualization of the estimated densities of population effects, illustrating the greater heterogeneity of the MA as compared to the MLR).

**Table 2.** $\hat{\mu }$: Average effect size (*SMD*), as estimated in a meta-regression model containing both sources. % effects > 0: Estimated percentage of positive population effects, as estimated in a meta-analysis or meta-regression model containing one source. % effects > 0.2: Estimated percentage of population effects stronger than *SMD* = 0.2. Discrepancies are calculated by subtracting between each statistical measure in the MLR from that in the MA, such that positive discrepancies indicate larger effect sizes in MA. Bracketed values are 95% confidence intervals, which are model-based for the $\hat{\mu }$ measures (Hedges et al., 2010) and for differences in $\hat{\mu }$ between sources and are bootstrapped for the percentage measures and for all cross-model comparisons (Mathur & VanderWeele, 2020a, 2021b). Confidence intervals are omitted when they were not statistically estimable (i.e., for percentage estimates that were very close to 0% or 100%).

**Statistical measure**	**Unadjusted model**	**Moderated model**
$\hat{\mu }$ in MA	0.34 [0.22, 0.46]	0.32 [0.16, 0.47]
$\hat{\mu }$ in MLR	0.34 [0.27, 0.42]	0.35 [0.22, 0.47]
$\hat{\mu }$ discrepancy	−0.01 [−0.14, 0.13]	−0.03 [−0.2, 0.14]
% effects > 0 in MA	86 [83, 90]	88 [83, 92]
% effects > 0 in MLR	100 [96, 100]	100
% effects > 0 discrepancy	−14 [−17, −7]	−12 [−17, −8]
% effects > 0.2 in MA	64 [61, 73]	71 [62, 79]
% effects > 0.2 in MLR	89 [76, 100]	100
% effects > 0.2 discrepancy	−25 [−40, −6]	−29 [−38, −21]

**Figure 2.**
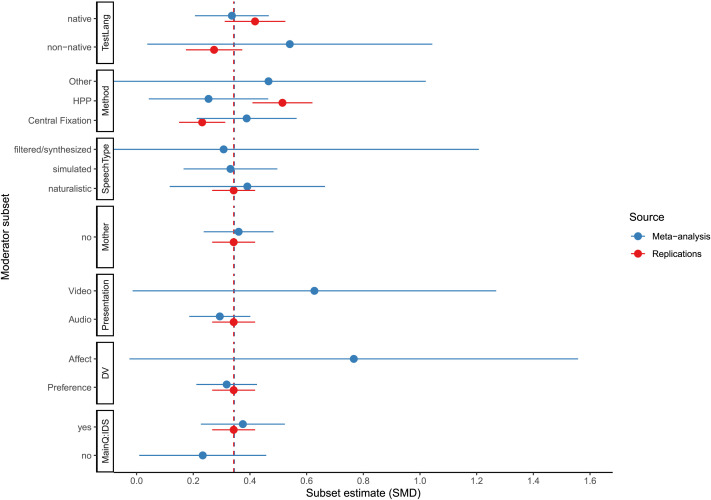
Forest plot showing, for each categorical candidate moderator, the pooled point estimates for the subset of studies in the MA and in the MLR, respectively, with a given level of the moderator (including only levels with at least 5 observations). Error bars are 95% confidence intervals. Error bars for many estimates are wide due to a limited number of observations at certain levels of a given moderator variable. Dashed vertical lines are unadjusted estimates in all MA studies and in all MLR studies. These lines overlap because the two estimates are virtually identical.

To delve further into the moderator analyses, Figure 2 shows, for each categorical candidate moderator, the pooled point estimates for the subset of studies in the MA and in the MLR, respectively, within a given level of the moderator. These simple, post hoc subset analyses stratify on only one moderator at a time and exclude those subsets that could not be estimated (e.g., familiarity of the speaker).

### Combined Models

We next considered models combining both the MA and the MLR datasets. We first fit an unadjusted model that combined the two sources without any additional moderators, confirming that effect sizes in the MA did not differ on average from effect sizes in the MLR, −0.01 (95% CI: [−0.14, 0.13]) units on the *SMD* scale (Table 2). There was considerable residual heterogeneity (estimated standard deviation of population effects $\hat{\tau }$_unadjusted_ = 0.27). Next, we fit a moderated model that explored whether IDS preference varied as a function of a set of theoretically meaningful predictor variables. The moderated model converged when we included three moderators besides source: infant age, test language, and method. Table 3 summarizes the estimates of the meta-regression for those remaining moderators. The estimated average effect size in the MA and in the MLR when setting the moderators to their average value (in the case of the continuous moderator infant age) or their most common value (in the case of the two categorical moderators; method: central fixation, test language: native) in the MA was, respectively, 0.32 [0.16, 0.47] and 0.35 [0.22, 0.47]. Thus, we also did not observe a significant difference between the effect sizes estimated for the MA and the MLR when controlling for moderators of theoretical interest, −0.03 [−0.20, 0.14]. Moreover, none of the three moderator variables showed a significant effect on the magnitude of IDS preference across the MA and the MLR. The residual heterogeneity increased slightly relative to the unadjusted model ($\hat{\tau }$_mod_ = 0.30). Overall, IDS preference was estimated to be stable across the data source, method, test language, and infant age. However, many of the confidence intervals were wide, indicating substantial uncertainty about moderation strength.

**Table 3.** Meta-regression estimates of moderation by various study design and participant characteristics. Intercept: estimated mean SMD when all listed moderators are set to 0 (for continuous moderators, the average value in the MA or, for categorical moderators, the most common value in the MA). The estimate of the categorical factor Meta-Analysis represents the change in *SMD* when this factor is true vs not. For infant age, the estimate represents the increase in effect size associated with a 1-month increase in mean infant age. For categorical moderators, estimates represent the increase compared to the reference level (Test Language: Native, and Method: Central Fixation, respectively). Bracketed values are 95% confidence intervals. *p*-values represent tests of moderators’ coefficients themselves (vs. 0) in the meta-regression.

Moderator	Est	CI	*p*-value
Intercept	0.35	[0.22, 0.47]	< 0.0001
Source: Meta-Analysis	−0.03	[−0.2, 0.14]	0.709
Infant Age (months)	0.01	[−0.00, 0.03]	0.120
Test Language: Non-native	−0.06	[−0.20, 0.09]	0.427
Test Language: Other	−0.17	[−2.68, 2.34]	0.544
Method: HPP	0.04	[−0.13, 0.21]	0.623
Method: Other	0.28	[−1.86, 2.42]	0.402

**Figure 3.**
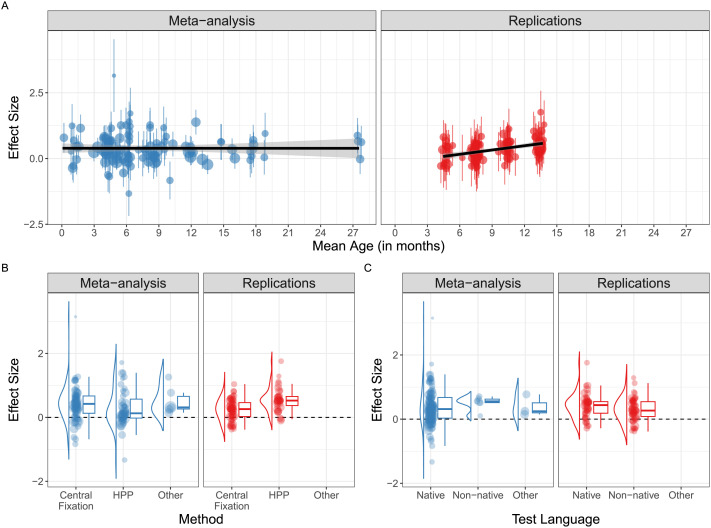
Overview of the distribution of effect sizes in the meta-analysis (MA) and replications (MLR) for three key moderators: infant age (A), method (B), and test language (C). In (A), the black line represents a linear fit through the effect sizes for each source and error bars for individual estimates are 95% confidence intervals.

Finally, we conducted an exploratory analysis in which we included the two-way interaction between source (MA vs. MLR) and each of the three moderator variables (infant age, test language, and method). The results from this model are summarized in Table 4. We found evidence for two key interactions. First, there was a significant interaction between source and infant age (*b* = −0.04 [−0.07, −0.02]; *p* = 0.002). This interaction was driven by the fact that there was a robust increase in the magnitude of the IDS effect across infant age in the MLR (*b* = 0.04 [0.02, 0.07]; *p* = 0.0004), but no appreciable change in IDS across infant age in the MA (*b* = 0 [−0.02, 0.02]; *p* = 0.82; Figure 3A). Second, we found a significant interaction between source and method (*b* = −0.38 [−0.63, −0.12]; *p* = 0.005). Here, the interaction appeared to be driven by a stronger effect of HPP (vs. other methods) in the MLR (*b* = 0.24 [0.13, 0.34]; *p* < 0.0001), but a numerically opposite, though not significant, effect of method in the MA (*b* = −0.14 [−0.39, 0.11]; *p* = 0.24; Figure 3B). There was no interaction between test language and source (*b* = −0.18 [−0.52, 0.15]; *p* = 0.21); however, both of these confidence intervals are wide, so moderate to strong moderation effects cannot be ruled out. Residual heterogeneity remained substantial ($\hat{\tau }$_mod_ = 0.27) but was reduced relative to the combined moderated model. We also assessed the robustness of the results by restricting the MA only to studies with average ages within the range observed in the MLR (3- to 15-month-old infants). We found broadly comparable results for the interaction between source and method and source and age, albeit with increased uncertainty (see Section 5.4.7 of the Supplementary Materials).

**Table 4.** Meta-regression estimates of the moderator interaction model. Intercept: estimated mean SMD when averaging across all (centered) moderators. Age (in months) is mean-centered. Test Language (Native vs. Other) and Method (HPP vs. Other) are treated as binary variables and centered. Bracketed values are 95% confidence intervals.

Moderator	Est	CI	*p*-value
Intercept	0.34	[0.25, 0.42]	< 0.0001
Source (centered)	0.03	[−0.14, 0.20]	0.656
Age (months; centered)	0.02	[0.01, 0.04]	0.001
Test Language (Native vs. Other; centered)	0	[−0.17, 0.17]	0.973
Method (HPP vs. Other; centered)	0.05	[−0.08, 0.17]	0.467
Source * Age	−0.04	[−0.07, −0.02]	0.002
Source * Test Language	−0.18	[−0.52, 0.15]	0.214
Source * Method	−0.38	[−0.63, −0.12]	0.005

### Publication Bias

We also considered the extent to which publication bias may be affecting estimates of differences between the MA and MLR. The MA contained 41 affirmative (i.e., statistically significant and positive-signed) and 71 nonaffirmative studies. We began by implementing a correction for publication bias, estimating the selection ratio from the MA itself. Based on the MA, we estimated that affirmative results were favored by a factor of 1.5. The average effect size in the MA after correction was *SMD* = 0.28 [0.14, 0.41]; *p* < 0.0001 (Vevea & Hedges, 1995), which was indeed somewhat smaller than the uncorrected estimate of *SMD* = 0.35. Next we applied sensitivity analyses for publication bias, considering what the true effect size would be under several different scenarios. Under hypothetical worst-case publication bias (i.e., if “statistically significant” positive results were infinitely more likely to be published than “nonsignificant” or negative results), the MA mean would decrease to 0.09 [−0.01, 0.18], which was significantly less than the estimate in the MLR. Under “typical” publication bias in this field (favoring affirmative results by 4.7-fold), the MA average would decrease to 0.17 [0.06, 0.27]. In both cases these estimates were lower than those in the MLR and—in the worst-case scenario—included zero in the 95% CI. Thus the estimate obtained in the MLR is—if anything—likely to be larger than the estimate of the MA under typical or worst-case assumptions about the severity of publication bias (cf., Section 5.4.5 of the Supplementary Materials for additional analyses of publication bias).

## DISCUSSION

Infant-directed speech (IDS) and its captivating nature for infants is an important phenomenon for many theories of early linguistic and social development. To improve our understanding of IDS preference and its boundary conditions, we compared and synthesized the evidence from two data sources: a community-augmented meta-analysis (MA) and an extensive multi-lab replication (MLR). Our analyses showed that the overall estimates across the two studies were similar, though the MA exhibited a greater degree of heterogeneity than the MLR. The estimates for the MA and the MLR remained comparable when including a range of theoretically-motivated moderators: adding moderators neither decreased heterogeneity nor produced significant differences between the MLR and MA estimates. However, in exploratory analyses, we found that the predicted effects of key moderators differed between data sources. Specifically, an interaction model showed i) an age-related increase in the strength of the effect in the MLR and no clear developmental change in the MA and ii) a stronger effect for the HPP method (compared to other experimental methods) in the MLR, but not in the MA. Together, these findings show that the MA and MLR provide converging evidence for the IDS preference across a wide range of participant, stimulus, and design characteristics, while also highlighting areas where substantial uncertainty remains about the effect of key moderators on IDS preference.

### Implications for Understanding the IDS Preference

Our main finding is that the IDS preference effect generalizes across relevant study dimensions in both the MA and MLR. The moderated models showed convergent results for IDS preference, with infants showing a general preference to attend to IDS over ADS stimuli during early development across a wide variety of ages, task contexts and linguistic backgrounds. This analysis thus conformed to previous studies showing that the unique properties of IDS robustly captivate infants’ attention from an early point in development (Cooper & Aslin, 1990; Fernald & Kuhl, 1987; Pegg et al., 1992; Werker & McLeod, 1989). The size of the IDS preference was also remarkably similar between the MA and the MLR: both data sources converged on an average effect size estimate of *d* ≈ 0.35. The convergence of the estimate across the two sources of evidence in such a broad range of conditions can aid developmental scientists in sample size calculations for future studies of IDS preference (Lakens, 2022). For example, the effect size estimate of *d* = 0.35 implies that a sample at least as large as *N* = 66 infants is needed to have 80% or greater power to detect an IDS preference at an alpha level of .05 in a within-participant design using a paired-samples *t*-test. Our full dataset is also openly available, allowing researchers to account for potential sources of variability and tune their power estimates to specific methodological and modeling choices.

Why does IDS exert such an early, widespread effect on infants’ preferential attention? One promising explanation posits that the engaging features of IDS reside in the mutual feedback loops between infant and caregiver, where infants’ active participation and caregiver responsiveness both contribute to the developmental process (Ko et al., 2016; Warlaumont et al., 2014). Given that adults use IDS as a consistent signal in addressing children during development, infants may start to associate the acoustic features of IDS with relevance and to recognize themselves as recipients of these salient utterances (Nencheva et al., 2021). This elevated attention to the speech stream, in turn, may drive the commonly observed language benefits of IDS during development (Golinkoff et al., 2015; Hartman et al., 2017; Peter et al., 2016).

At the same time, our exploratory analysis also found critical points on which the evidence from the MA and the MLR disagreed: infant age and experimental task showed distinct effects across the two sources. The different developmental trajectories of the IDS preference effect paint a complicated picture of the role of IDS during development. The linear increase with infant age in the MLR conforms to evidence that the IDS preference grows in response to experience with positive social interactions and increased participation in communicative exchanges (Ko et al., 2016; Warlaumont et al., 2014). On the other hand, the finding of stability across infant ages in the MA—which has also been previously reported in individual, smaller-scale studies in the literature (Newman & Hussain, 2006; Segal & Newman, 2015)—may indicate that IDS continues to be similarly relevant throughout early development.

The conflict in developmental trajectories in the MA and MLR may be driven by factors other than the underlying construct. For example, as discussed in the original ManyBabies 1 paper (The ManyBabies Consortium, 2020), the speech stimuli may have been best suited for the older age ranges in the study, or older infants may have exhibited more measurable behavioural responses. This would also accord with evidence that some acoustic characteristics of IDS change as children grow older (Cox et al., 2023). Conversely, in the MA, investigators had the freedom to tailor their stimuli and methods to the particular infant age investigated. One potential consequence of researchers tailoring methods to maximize effect sizes within the studied age range is that this practice may mask age-related changes in the strength of the IDS preference effect. This discrepancy between the results of the MA and MLR are not easily resolved. One way to improve our understanding of the developmental trajectories of the IDS preference would be to conduct more experiments on how infant looking time measures relate to their experience of the underlying construct (Kosie et al., 2023), and to use other higher-resolution non-behavioural measures to triangulate the effects that modulate infants’ IDS preference (e.g., Nencheva et al., 2021).

The finding that experimental task produced diverging results across the MA and MLR again demonstrates limitations in the conclusions we can draw from each source on its own. For example, as discussed in the original paper (The ManyBabies Consortium, 2020), the finding of a stronger estimate in the MLR for studies using the HPP may be a function of the greater effort required on the part of the infant in the task, leading to stronger engagement and therefore to stronger effects. However, the MA did not demonstrate larger effect sizes for HPP methods, and at least numerically, the effect was in the opposite direction (see Figure 3). Smaller effect sizes for HPP compared to central fixation aligns with previous meta-analytic results in the infant literature (Bergmann et al., 2018). Taken at face value, these results call into question the generalizability of the result from the MLR. However, both the MLR and MA involved data from studies that self-selected the methodology employed to test the effect, severely limiting the causal inferences that can be drawn about the effect of methodology on IDS preference.^4^ Future large-scale MLR studies may benefit from conducting random assignment of experimental methodology to participating labs; this experimental design would provide valuable information about the importance of methodological choices, the relation between MLRs and MAs, as well as how to interpret findings from infant studies more generally.

Our exploratory interaction analyses showed no robust differences in the effect of native language across the two sources of evidence. These results are consistent with the hypothesis that the main captivating features of IDS may reside in acoustic properties that are commonly attested across distinct languages (Cox et al., 2023; Hilton et al., 2022). We should note, however, that this result may have been driven in part by the unbalanced nature of the MA data, where only 5.4% of the effect sizes (vs. 54.9% in the MLR) included infant looking times to non-native speech stimuli. In the full sample of the original MLR (The ManyBabies Consortium, 2020), monolingual infants acquiring North American English had a stronger preference to attend to North American English IDS than monolinguals acquiring another language. The results here may thus be driven primarily by the imbalance in the MA effect sizes as well as the subsample characteristics of the MLR. This interpretation would also be in line with evidence from another recent MLR (Byers-Heinlein et al., 2021) showing that bilingual infants with a higher percentage of exposure to North American English had a stronger North American English IDS preference.

The overrepresentation of North American English in the MLR and especially in the MA is emblematic of the substantial language bias in developmental research (Christiansen et al., 2022; Kidd & Garcia, 2022; Kidd et al., 2023) and in IDS research in particular (Cox et al., 2023; Cristia, 2023; Ochs & Schieffelin, 1984). Oversampling from particular populations severely constrains our understanding of the global variability in the use of IDS across languages, dialects and cultures (Casillas et al., 2020; Cristia, 2023; Floccia et al., 2016), and this in turn limits our ability to construct generalizable theories about the features and functional relevance of IDS in different cultural settings. Based on the cumulative findings presented here (see Table 1), future research on IDS preference should focus on expanding language diversity both with respect to participants’ language backgrounds and the speech stimuli tested, in order to evaluate the generalizability of the results to other sample characteristics (e.g., Tsui et al., 2023) and first languages (e.g., Soderstrom et al., in prep).

The complex interactions between sample characteristics in both the MA and MLR also highlight an important limitation in our conclusions: scarcity of available data on moderator interactions can hinder attribution of variation and accurate estimation in statistical models (Lipsey, 2003; Tipton et al., 2019). For example, all of the studies using artificial stimuli in the MA use a method that is neither HPP or central fixation, severely limiting the inferences we can draw about the effects of this stimulus type. This paper thus emphasizes the need for careful consideration and comprehensive assessment of moderator variables in future research to better understand and reconcile results across individual studies as well as MLRs and MAs (cf. Figure 1 in Section 4.2 of the Supplementary Materials). In the current context, theory-driven investigation of the extent to which the IDS preference effect is modulated by cross-linguistic variability in IDS features as well as differences in language exposure will be an important topic for future research.

### Implications for the Relationship Between MAs and MLRs

Overall, both MLRs and MAs are useful techniques to combine and synthesize evidence from multiple studies. Each technique, however, has benefits and drawbacks. If used critically and with an understanding of its inherent limitations, MAs can serve as a crucial tool to assess the progress of a field, to highlight its strengths and weaknesses, to provide methodological recommendations, and to offer directions for future research endeavors (Fusaroli et al., 2022; Nguyen et al., 2022). An inherent limitation of MAs, however, is that the data are filtered through the publication process. This process acts as a bias that selects for statistically significant findings, typically leading to an inflation of effect sizes in the MA (Kvarven et al., 2020; Lewis et al., 2022). Notably, however, our worst-case publication bias estimates for our MA were in fact *lower* than the MLR estimate, suggesting that estimates of the IDS preference phenomenon might not suffer from the same degree of publication bias as other phenomena in the developmental literature. MAs have also come under scrutiny for reasons beyond publication bias, including a lack of reproducibility and errors in the extraction of data (Maassen et al., 2020). MAs may be particularly susceptible to errors as they adopt any errors in the original studies (see e.g., Nuijten et al., 2016), combined with any new errors introduced by the MA. In the current paper, we found substantial errors in the original MA (Dunst et al., 2012), which changed the interpretation of some of the results (cf. Section 2.1 in the Supplementary Materials for a full list of revisions to the original MA; Section 5.3 for an overview of results across the original, revised and community-augmented datasets; and Section 6 for in-depth discussion of these discrepancies and our rationale in focusing on the community-augmented MA). In consideration of these limitations—including errors in reporting effect sizes, data curation errors, and omission of reported effect sizes—we call for higher standards in transparency of all steps of the meta-analytic process (Tsuji et al., 2014). These may fruitfully be pursued within already established open science initiatives for meta-scientific endeavours (e.g., MetaLab, https://metalab.stanford.edu/).

MLRs, on the other hand, can provide an estimate of the phenomenon of interest that is free from publication bias, but within a relatively restricted range of stimuli and methodological designs and with a very high cost in time and money. In the current context, individual labs were themselves allowed to select experimental methodology. Crucially, this limits the degree to which we can make causal inferences about the effect of methodology. One possible step that future MLRs could consider is randomly assigning participants to key moderators of interest (such as specific methodological choices). Manipulating a wider variety of moderators systematically would allow for stronger causal inferences and could lay the groundwork for a fuller understanding of the moderating role of design choices in the investigation of key phenomena.

### Conclusions

In summary, we find robust evidence that IDS captivates infants’ attention during development across two sources of evidence: a community-augmented MA and a MLR. Synthesizing the evidence from these two sources allowed us to show that IDS preference generalizes across a broad range of participants, ages, methods, and stimuli, albeit with substantial remaining uncertainty about how the magnitude of the IDS preference effect varies across key moderators. Many key questions about the IDS preference effect remain open. Evidence between the MLR and MA conflicts with respect to the developmental trajectory of IDS preference and the degree to which different methodologies elicit varying effect magnitudes. Overall, this study shows that MAs and MLRs provide distinct but complementary approaches to assessing phenomena and the factors that modulate them: MAs allow for estimating effects across heterogeneous design choices and populations in the extant literature, while MLRs offer an approach for large-scale, high-precision estimation of key effects within similar implementations and free from publication bias. Rather than considering either MAs or MLRs as the gold standard, this work demonstrates how integrating each of these two sources of evidence offers an attractive path forward for building cumulative evidence in psychological science.

## ACKNOWLEDGMENTS

The funders had no role in the design, conduct, or reporting of this research. We would also like to thank the following research assistants: Lucy Anderson, Stephen Gilliat, Heewon Hwang, Sarah Kamhout, John Muldowney, and Taylor Orr.

## FUNDING INFORMATION

This research was funded by SSHRC Partnership Development Grant GR019187 to MS. MBM was supported by NIH R01 LM013866-01. MZ was supported by a grant from the Eunice Kennedy Shriver National Institute of Child Health & Human Development of the National Institutes of Health under Award Number F32HD110174. Jessica Kosie was supported by NSF SBE Postdoctoral Research Fellowship 2004983 and NIH F32 F32HD103439.

## AUTHOR CONTRIBUTIONS

The following lists each author’s contribution to this paper based on CRediT (Contributor Roles Taxonomy). An overview of authorship contributions can be viewed here: https://docs.google.com/spreadsheets/d/1CQfw_ASSMT5boxpNGSfFmJvt8KQ0yiePgpwDEbRIrY0/edit?usp=sharing.

Martin Zettersten: Conceptualization, Data curation (lead), Data collection – coding papers (lead), Documentation, Formal analysis, Project administration, Software, Validation, Visualization, Writing – original draft (co-lead), Writing – review and editing (co-lead). Christopher Cox: Conceptualization, Data Curation, Formal analysis, Project administration, Software, Validation, Visualization, Writing - original draft (co-lead), Writing – review and editing (co-lead). Christina Bergmann: Conceptualization (lead), Data curation, Data collection – coding papers, Documentation, Formal analysis, Project administration, Software, Writing – original draft (co-lead), Writing – review and editing. Melanie Soderstrom: Writing – original draft, Writing – review and editing. Angeline Sin Mei Tsui: Conceptualization, Data collection – coding papers, Documentation, Writing – review and editing. Julien Mayor: Conceptualization, Writing – review and editing. Rebecca A. Lundwall: Data collection – coding papers, Resources, Writing – review and editing. Molly Lewis: Data curation, Formal analysis, Software. Jessica E. Kosie: Conceptualization, Data collection – coding papers, Data curation, Documentation, Writing – review and editing. Natalia Kartushina: Conceptualization, Data collection - coding papers, Data curation, Documentation, Writing – review and editing. Riccardo Fusaroli: Conceptualization, Software, Validation (lead), Writing – review and editing. Michael C. Frank: Conceptualization, Visualization, Writing – review and editing. Krista Byers-Heinlein: Conceptualization, Software, Visualization, Writing – original draft, Writing – review and editing. Alexis K. Black: Conceptualization, Data collection – coding papers, Data curation, Documentation, Writing – original draft. Maya B. Mathur: Conceptualization, Formal analysis (lead), Software (lead), Validation, Visualization (lead), Writing – original draft (co-lead), Writing – review and editing.

## DATA AVAILABILITY STATEMENT

All code, materials, and data required to reproduce this research are publicly available and documented on OSF (https://osf.io/amj7u/) and GitHub (https://github.com/christinabergmann/IDSPreference_ManyBabiesMeta/).

## Notes

^1^ For parallel analyses using both the original meta-analysis and a revised version of the meta-analysis, as well as a discussion of discrepancies, see Supplementary Materials (Section 5 and Section 6).^2^ We use the term “population effects” to refer to population parameters, rather than to point estimates with statistical error.^3^ We used the packages boot (Davison & Hinkley, 1997), table1 (Rich, 2021), MatchIt (Ho et al., 2011), xtable (Dahl et al., 2019), Matrix (Bates & Maechler, 2021), ggplot2 (Wickham, 2016), stringr (Wickham, 2019), forcats (Wickham, 2021a), tidyr (Wickham, 2021b), scales (Wickham et al., 2020), readr (Wickham & Hester, 2020), dplyr (Wickham et al., 2021), testthat (Wickham, 2011), fastDummies (Kaplan, 2020), weightr (Coburn & Vevea, 2019), tableone (Yoshida & Bartel, 2020), renv (Ushey & Wickham, 2021), here (Müller, 2020), tibble (Müller & Wickham, 2021), purrr (Wickham & Henry, 2020), report (Makowski et al., 2023), data.table (Dowle & Srinivasan, 2020), corrr (Kuhn et al., 2020), PublicationBias (Braginsky et al., 2023), metafor (Viechtbauer, 2010), tidyverse (Wickham et al., 2019), knitr (Xie, 2014), and robumeta (Fisher et al., 2017).^4^ We should note that the goal of the MLR was not to replicate a single study, but rather to investigate how well the IDS preference generalized across different laboratories and methods. Because self-selection of methodologies likely varies systematically with other characteristics particular to each laboratory and study, we can at best make tentative conclusions about the effect of methodology on infants’ IDS preference.

## Supplementary Material


