# Supplementary material for: Evidence for Infant-directed Speech Preference Is Consistent Across Large-scale, Multi-site Replication and Meta-analysis
Source: Open Mind (Camb). 2024 Apr 3;8:439–61. doi: 10.1162/opmi_a_00134 (PMC11045035; doi:10.1162/opmi_a_00134)
Supplement: Supplementary file 1 [file opmi-08-439-s001.pdf]

Supplementary Materials: Evidence for infant-directed speech preference is consistent across large-scale, multi-site replication and meta-analysis

Contents

1 Preregistration **2**
1.1 Preregistration Approach 2
1.2 Changes and additions to preregistered protocol 2

2 Revising and augmenting the meta-analysis **3**
2.1 Revisions to Dunst et al. (2012) 3
2.2 Augmenting the meta-analysis 6

3 Methodological details for ManyBabies 1 **6**
3.1 Sampling 6
3.2 Exclusion Criteria 6

4 Moderators **7**
4.1 Moderator Descriptions 7
4.2 Moderator Distributions 9

5 Supplementary Results **10**
5.1 Results from the uncorrected meta-analysis 10
5.2 Results from the **revised** Dunst et al. (2012) meta-analysis 13
5.3 Overview of Results across Meta-Analytic Datasets 20
5.4 Additional analyses with the community-augmented meta-analysis 20
5.4.1 Overview over included studies and effect sizes in the MA 20
5.4.2 Overview over included studies and effect sizes in the multi-lab replication 22
5.4.3 Estimated densities of population effects in the MA and MLR 25
5.4.4 Sensitivity analysis: Within-subjects experiment design 26
5.4.5 Additional analyses supporting publication bias methods 26
5.4.6 Exploratory sensitivity analysis: Applying more stringent participant inclusion criteria in the multi-lab replication 28
5.4.7 Exploratory sensitivity analysis: Restricting the age range when investigating interactions between the MA and MLR 28

6 Discussion of discrepancies between results across MA datasets **30**

1. Preregistration

1.1. Preregistration Approach

All confirmatory analyses were preregistered prior to data analysis (`https://osf.io/scg9z?view_only=7fd9e41122e042cfa998e50cf0336572`). Given that all analyses involved preexisting datasets, we outline how the team approached accessing and curating the data in relation to the development of the preregistration protocol below. We wrote the preregistration protocol after we had accessed the original MA dataset (a digitized version of information in the Appendices of Dunst et al. (2012)), assembled the replication data (via a public Github repository at https://github.com/manybabies/mb1-analysis-public), and conducted basic cleaning on both datasets but before conducting any analyses relevant to the preregistered research questions. During the process of developing and planning statistical analyses, the statistician (MBM) was provided with only a "dummy" version of the combined dataset (comprising both the MA and MLR data) in which the point estimates and their variances had been randomly permuted across the two sources (i.e. the MA and the MLR). All authors co-developed the preregistration protocol, some of whom had access to the veridical dataset during protocol development but who had not conducted any of the planned analyses. Note that coauthors were aware of the main results of the original MA and the MLR as they were reported in the published reports (Dunst et al., 2012; The ManyBabies Consortium, 2020).

1.2. Changes and additions to preregistered protocol

Here, we describe and justify deviations from our preregistered protocol.

1. We focused all analyses on a revised and expanded community-augmented meta-analysis (`https://langcog.github.io/metalab`) instead of the original (Dunst et al., 2012) meta-analysis. Our preregistered plan was to conduct the meta-analysis by transcribing the dataset from (Dunst et al., 2012), with several additional moderator variables coded. However, over the course of the project, we revised and expanded the meta-analysis in two ways. First, we revised substantial issues discovered in the Dunst et al. (2012) meta-analysis (see Section 2.1 for details). Second, the meta-analysis was substantially augmented by the metalab community of infant researchers, leading to a significantly expended meta-analysis (30 papers, 112 effect sizes). We ultimately chose to focus our primary analyses on the community-augmented meta-analysis as the most comprehensive, accurate meta-analytic dataset on IDS preference available. All analyses with the original and revised Dunst et al. (2012) datasets are reported below (Section 5) and discrepancies between the datasets are discussed in more depth (Section 6).

2. Our preregistration specified that we would estimate the percentages of positive effects and of effects stronger than $SMD = 0.20$ for each source by using a single meta-regression model (Mathur & VanderWeele, 2021). However, the heterogeneity estimate in such a model is an average over the two sources, and it became apparent during data analysis that the MA showed considerably more heterogeneity than the MLR. We

therefore estimated the percentage metrics for each source by fitting separate meta-analysis or meta-regression models, as described in the main text. For the same reason, we omitted an analysis we had planned in which we would have estimated the difference in the heterogeneity estimates from the unadjusted model versus the moderated model, each containing data from both sources.

3. We added additional exploratory analyses to investigate the interaction between study-level moderators and the two data sources. These exploratory analyses were added to enable estimation of discrepancies between the two sources in terms of the effect of the three predictors (i.e., infant age, test language and method) on IDS preference. For this analysis, we simplified the test language predictor (Native vs. Other) and the method variable (HPP vs. Other) into centered, binary variables (as opposed to three-level categorical variables) in order to achieve model convergence. We describe this model as exploratory throughout the main manuscript.

2. Revising and augmenting the meta-analysis

2.1. Revisions to Dunst et al. (2012)

While coding additional moderators for the studies included in a previous meta-analysis of infant-directed speech preference (Dunst et al., 2012), we encountered substantial issues with the results and study classifications reported in the original meta-analysis. The main issues we identified are as follows.

- **incorrectly reported effect sizes.** The effect sizes for some papers were inconsistent with the effect sizes we determined based on reported statistics and figures in the original papers. In some cases, we could identify the source of the error (e.g., incorrectly treating the condition manipulation as between-subjects rather than within-subjects when computing effect size), while in other cases we were unable to trace the source of the incorrect effect size estimate in the original meta-analysis. (n=4 effect sizes from two studies corrected)

- **Inappropriate inclusion of experiments or study conditions.** Some experiments or study conditions were included incorrectly. For example, in one instance, the original meta-analysis treated conference papers or theses and corresponding journal articles as separate entries, despite the fact that these papers reported on the same data. In other cases, study conditions were included that did not represent a test of infant-directed speech preference. We also excluded one study that included a highly atypical dependent measure of IDS preference - manipulating a physical toy - that differed substantially from all other included studies. (n=6 effect sizes from three studies removed)

- **Inappropriate exclusion of experiments or study conditions.** For some papers, the original meta-analysis reported only a subset of the experiments or study conditions in a given paper that represented a test of IDS preference. For example, in one instance, a paper included 11 separate conditions evaluating IDS preference across all experiments,

but only 6 of these conditions were included in the original MA. (n=10 effect sizes from three studies added)

- **Inaccuracies or inconsistencies in moderator variables.** We also encountered many instances in which moderator variables were coded incorrectly or inconsistently across studies. For example, papers using the same stimulus set were sometimes coded as differing on a stimulus dimension (such as whether the speaker had experience interacting with children).

To address these issues, we sought to revise the MA to match the information in the original papers. A group of six coders inspected each individual paper, documented each issue they identified in the original MA, and proposed a solution to the issue. Then, at least one other coder reviewed the issue and discussed the best solution with the first coder. Any issues that involved a substantial change to an effect size estimate (due to an incorrect effect size, or due to including or excluding a particular study condition) were discussed and agreed upon by the entire coding group. Whenever possible, we re-computed effect sizes from information reported in the source paper. When the paper included insufficient information to derive an effect size estimate, we used the effect size reported in the appendix of the (Dunst et al., 2012) meta-analysis, so long as the effect size was not clearly inconsistent with the results reported in the original paper. Incorrectly included effect sizes were removed and inappropriately excluded effect sizes were included in the updated meta-analysis. In total, the effect sizes reported for 8 of the 16 studies in the (Dunst et al., 2012) meta-analysis were revised, with 10 effect sizes included in the original meta-analysis altered (6 removed and 4 corrected) and 10 new effect sizes from the original studies added in the revised meta-analysis (Table 1). All moderator variables were updated to reflect the information reported in the original paper. For a full overview of all issues we encountered during the re-coding process and each corresponding change that was made to the original meta-analysis, see `https://docs.google.com/spreadsheets/d/e/2PACX-1vQaFJkLsVlZNhj8-L8FJ3rmEkfKg5KHZALCMLrp9ki7Fbd9n5xhGGvLGsKQKB296gL8QlFIMq3c-nF7/pubhtml`.

Table 1: *Overview over the main revisions to Dunst et al. (2012)*

**Study ID**	**Main Issue**	**Solution**	**Explanation**	**Original No. Effects**	**Updated No. Effects**
Cooper1990	none	none	No major issues	2	2
Cooper1994	incorrect effect sizes and sample sizes	correct sample sizes/ design coding and recompute effect sizes based on means and SDs extracted from the figure	Most of the key issues relate to Experiment 3, which was previously incorrectly coded/ handled as a between-subjects condition and where sample sizes were incorrect (20 *total* infants, split between an IDS-first and ADS-first group, so n=10 per row), leading to some likely inaccurate effect size estimates. The sample size values have been corrected and means/ SDs were extracted from the figures in order to recompute the effect sizes.	4	4
Cooper1997	none	none	No major issues	3	3

Fernald1985	incorrect effect sizes in Dunst	recomputed effect sizes	Effect size reported by Dunst is d=0.65. However, after extracting data based on Figure 3, the effect size was recomputed as d=0.32	1	1
Fernald1987	incorrect effect sizes in Dunst	recomputed effect sizes	The effect sizes for all three experiments reported in Dunst are incompatible with the data reported in the paper. Original data was determined based on Ferrnald's dissertation (Figure 5) and effect sizes were recomputed in each case	3	3
Glenn1983	atypical procedure and implausible effect sizes	removed experiment	Two main issues: (a) the procedure is highly unusual and is not a looking-time-based procedure (unlike every other study in the meta-analysis). Therefore, the study should be excluded based on its procedure type. (b) The effect sizes for this study are extremely large (ds≈2.5), and insufficient information is provided in the original paper to determine how they were computed by Dunst. Given these two major issues, we decided to exclude this study.	2	0
Kaplan1995a	none	none	Only minor issues not directly related to effect size (though effect size cannot be recomputed from data available in the paper)	2	2
Kaplan1995b	removed effects from trial 10	removed data points from experiment	Audio (IDS/ ADS) was presented only on trial 9, hence only data from this trial is included. Three subsequent trials (trials 10, 11 and 12) are presented in silence. Dunst included the first of these (trial 10) - however, it is not clear why trials 11 and 12 were not also included. In our view, there are two defensible positions: include only trial 9 (only trial with audio stimulus), or all trials 9-12 (post-audio). We think the former option is the most straightforward (include only trial 9)	4	2
Pegg1989	duplicate study	removed experiment	This paper is a conference proceedings paper using data that was eventually published in Pegg1992 (with a high, high likelihood). We therefore included only Pegg1992 and removed Pegg1989.	2	0
Pegg1992	none	none	One key issue cannot be resolved (cannot determine non-significant effect sizes in Exp 1)	2	2
Schachner2011	none	none	Only minor issues not directly related to effect size	2	2
Singh2002	missing studies	add previously omitted studies testing IDS/ ADS preference	Experiments 1, 4, and 5 were previously not included in the meta-analysis, despite testing an IDS vs. ADS difference	6	11
Singh2009	missing conditions	add previously omitted condition testing IDS/ ADS preference	Comparison of unfamiliar passages previously not included, despite also providing a test of IDS vs ADS preference	2	3
Trainor1996	missing conditions	split data into six individual between-subjects conditions	Previous coding did not distinguish between different conditions of the experiment and appears to have omitted conditions with effects in the opposite direction, leading to an inflated effect size estimate; solution is to code each of the six conditions (each presenting a different IDS/ ADS stimulus set) as separate rows/ effect sizes	2	6
Werker1989	none	none	One key issue cannot be resolved (cannot determine non-significant effect size in Exp 3)	6	6

Werker1994	none	none	No large issues related to effect size. Effect sizes will be recomputed from Ms/ SDs derived from figures	8	8

2.2. Augmenting the meta-analysis

After the meta-analysis data from Dunst et al. (2012) had been digitized for use on Meta-Lab (`https://langcog.github.io/metalab`), it was open to community-augmentation (i.e. members of the community could propose additional relevant papers). These papers were screened and added by a data manager. Additionally, experts from the field could suggest papers to add. In addition to ad-hoc additions, new studies were primarily added in two new literature search waves conducted in 2017 and 2019 in Google Scholar using a reverse-citation approach, identifying all publications citing two early studies of IDS (Fernald (1985); Cooper & Aslin (1990). Studies were screened for inclusion by trained community members. The final set of studies were coded by the same members of the authorship team who also conducted the revision of the Dunst et al. (2012) meta-analysis (Section 2.1) to ensure continuity in the coding process. All papers were coded by at least two team members. All coding discrepancies were documented and resolved through discussion among the entire coding team. Overall, this process resulted in a community-augmented meta-analysis comprising 30 studies contributing a total of 112 estimates.

3. Methodological details for ManyBabies 1

3.1. Sampling

Over the course of 14 months, labs were asked to test infants in up to four age groups (3-6, 6-9, 9-12, 12-15 months of age) and contribute at least 16 potentially eligible participants (before experimental exclusions, such as not enough data). Participants were tested across four continents (North America, Europe, Asia, Australia), and grew up learning one of 12 different languages, and therein four different varieties of English; of which two were classified as North-American (Canadian and US English) and 2 as non-North American English (Australian and British English). Since the stimuli were in North American English, only North American English learning participants were considered to be listening to native speech. All participants were monolingual; we are not including the data from the bilingual sample sister project (Byers-Heinlein et al., 2021).

3.2. Exclusion Criteria

Participant exclusion criteria included: (1) younger than 3 months or older than 15 months; (2) a known developmental delay; (3) premature birth (before 37 weeks); (3) experimenter error; (4) no usable trials (less than 1 trial per condition with at least 2 s total looking time to the screen). Trials were excluded (1) when the minimal looking criterion of 2 s was not met or the infant was inattentive/fussy during the trial; (2) due to technical errors; and (3) because of parental interference. Our dataset has all trial-level exclusions already applied

and thus follows the published report; for detailed exclusion statistics we refer to the paper reporting on the replication (The ManyBabies Consortium, 2020).

4. Moderators

4.1. Moderator Descriptions

The moderators included in our confirmatory analyses vary in their theoretical importance. We discuss them in the order reflecting the expected magnitude of impact they have on infant performance. Note that the MLR only varies in the first three of these moderators. We were only able to successfully fit models including these first three moderators, as reported in the main text.

Age Age is a key factor in developmental phenomena, which often emerge and change over time. Both the MLR and MA report a positive age effect, such that older infants show a larger preference. Theoretically, age could affect the measured preference for IDS in various directions. Younger infants might be expected to show increased preference for IDS due either to greater focus on broad acoustic characteristics of the speech or due to the greater importance of IDS pedagogically early in development. However, older infants become more mature language processors and accumulate language experience, which might allow them to more easily "tune in" to features of IDS. Older infants are also more cognitively and physically mature and might logically be expected to "perform" better in laboratory experiments more generally for reasons unrelated to the specific phenomenon under investigation (e.g., attentional control, comprehension of social expectations) (see e.g., Bergmann et al., 2018).

Test language While there are reasons to believe that some characteristics of IDS are universal, there is variation in their realization in different languages (Fernald et al., 1989; Cox et al., 2022). As a consequence, infants' preference for IDS may vary depending on whether or not stimuli were presented in infants' native language. Furthermore, even if IDS itself were universally specified, infants hearing speech stimuli in a non-native language may devote attentional resources differently than those hearing speech in their native language. Indeed, ManyBabies 1 reports that the IDS preference is stronger when the stimuli matched infants' native tongue.

Experimental method Method effects have been shown across tasks and ages, for example when pooling over 12 meta-analyses on early language acquisition (Bergmann et al., 2018). We thus expect an effect of method to be present in the aggregated data as well, among other factors because the original Dunst et al. (2012) MA was part of the pooled datasets for the just-cited meta-MA and because the current MLR found method effects. But whether these effects are consistent across datasets is unknown. We group method as follows: Headturn Preference Procedure (HPP), Central fixation (CF; including single-screen and eyetracking), Other (Forced Choice, FC; Conditioned Headturn, CHT). The categories are motivated by similarity in the tasks, i.e. either looking to vs away from a single screen with an unrelated visual display (CF), turning the head to the side towards flashing lights (HPP), or other tasks

which have only been used for a handful of estimates each (forced choice and conditioned headturn, both being used for four estimates, respectively). Note that the ManyBabies 1 study only included the first two of these three categories. ManyBabies 1 also included a third category, eye tracking. In the current analyses, we collapsed the methods termed "central fixation" and "eye tracking" in ManyBabies 1 into a single method category (CF), because we defined method in terms of the type of task procedure (as opposed to technical equipment used), and both central-fixation and eye-tracking experiments in ManyBabies 1 involved the same task procedure (i.e., a given trial worked exactly the same way from the perspective of an infant participant).

Speech type Conditions under which the stimuli were recorded were reported to influence effect size in the MA. This stimulus characteristic includes naturalistic (i.e. parents talking to their child), simulated (i.e. someone speaking *as if* talking to a child), and filtered or synthesized speech (i.e. manipulated recordings that sounded unnatural). As found in the original MA, we expect that the strength of the effect is highest for natural speech (used in the large-scale replication), followed by simulated speech, with filtered and synthesized speech showing smaller effects in turn. This effect might interact with age, but we did not include this interaction in the preregistered analyses because of power concerns and because we have insufficient grounds in the literature for strong predictions.

Speaker familiarity We expected a stronger effect for a highly familiar speaker, especially the infant's main caregiver (the infant's mother in the included studies). An advantage for maternal speech was reported by van Rooijen et al. (2019) and Barker & Newman (2004). These studies all compared the infant's own mother's voice to the voice of another infant's mother in the same study. In contrast to these experimental manipulations of speaker familiarity showing a benefit for the own mother's voice, however, Dunst et al. (2012) report a smaller effect size for the child's mother when comparing across studies. To further investigate this effect, we added whether the speaker was the child's own mother as a possible moderator.

Mode of presentation We tracked whether infants were presented with an unrelated visual stimulus or saw a video of a speaker, as this methodological variation might heighten infants' attention. This effect could either lead to an overall longer looking time across conditions (which would not be reflected in the effect size) or an increase in the difference between conditions (i.e. a larger effect).

Dependent measure Following the MA, we grouped the dependent variables into preference and affect. Studies either measured infants' looking time to a visual display (collapsing over the previous mentioned distinction between a related and unrelated visual stimulus) or infants' facial expression (e.g., smiling). All of these measures rely on behaviours that differ in the effort and conscious control the infant needs to exert (e.g., automatic smiling versus turning the head sideways and maintaining this position), and thus might impact the measured effect.

Study goal We coded whether infants' preference for IDS over ADS was the main research question of a paper since studies might also display the two types of speech stimuli to assess secondary phenomena, such as whether the presence or absence of IDS influences infants' preferences for specific speakers (Schachner & Hannon, 2011). While such studies contain the main comparison of interest, authors might add factors relevant to their research question, which in turn may lead to comparatively less controlled IDS and ADS stimuli. Since most stimuli are not available to us for direct comparison, we use the variable of whether IDS preference was a key research question as a proxy for stimulus quality.

4.2. Moderator Distributions

A key factor affecting our ability to fit moderator models is overall differences in the distribution of moderators between the MA and MLR, as well as differences in these distributions across multiple moderators (Tipton et al., 2019). To illustrate these differences, **Figure 1** shows the distribution of three key moderators (Infant Age, Method, and Test Language) for the MA and MLR. Notable patterns include: (1) only MA studies use artificial stimuli and all of these studies use methods that are neither HPP or CF; (2) MA studies using the HPP method exclusively test infants in their native language; and (3) MA studies have a wider age distribution, but this is especially true for studies using the CF method.

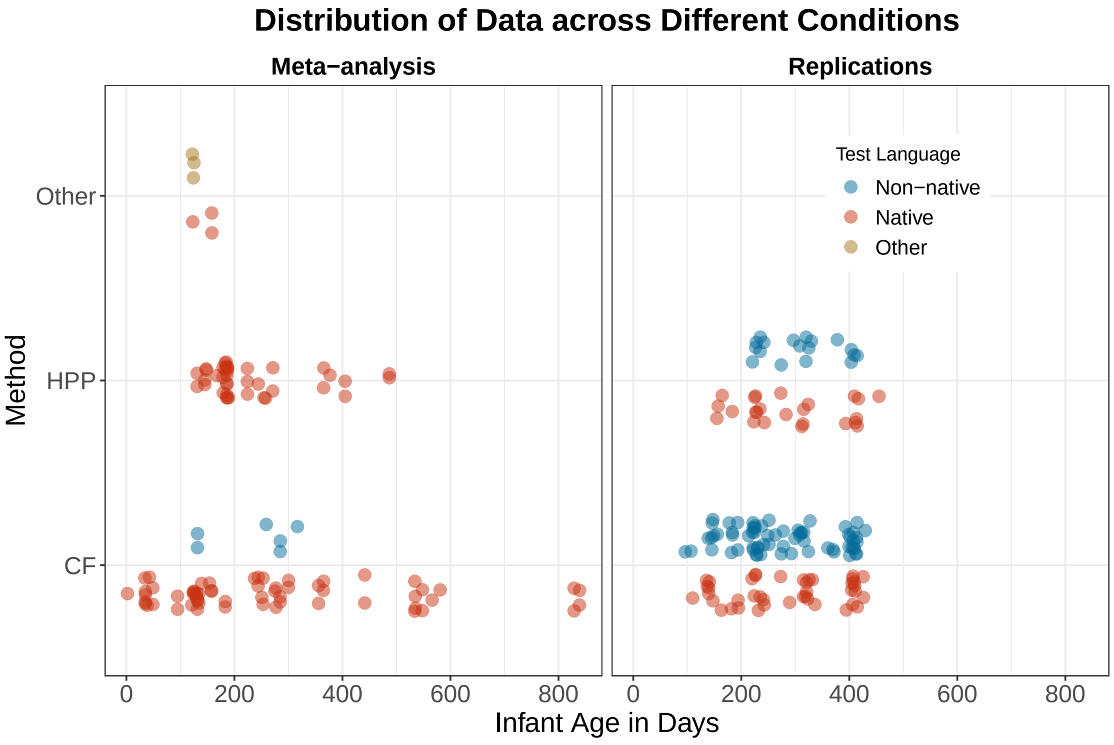

Figure 1: *Overview of distributions between moderators in the MA (left) and MLR (right). Studies depicted with a blue circle used non-native stimuli, studies depicted in orange used stimuli in infants' native language, and studies in brown used artificial stimuli.*

.

5. Supplementary Results

5.1. Results from the uncorrected meta-analysis

Based on our revision of the MA by Dunst et al. (2012) during coding of additional moderator variables, we noted several points of concern, such as the duplicate inclusion of data based on proceedings and journal papers (see section 2.1 in the Supplementary Materials for a full overview). Because our preregistration focused on the original (uncorrected) meta-analysis, we report statistical results for the original meta-analysis for transparency reasons; however, the results from this section should be treated with caution given the issues identified. The updated, community-augmented dataset in the main manuscript that includes corrections to the issues and errors in the original meta-analysis — as well as an updated literature search that more than doubles the number of individual effect size estimates (see Table 8 below) — provides the most up-to-date meta-analytic estimate of IDS preference.

The average estimated effect size for only the studies from the original, uncorrected Dunst et al. MA was 0.67 (95% CI: [0.38, 0.95]; $p < 0.001$), with considerable heterogeneity (estimated standard deviation of population effects $\widehat{\tau} = 0.51$). Nearly all of the population effects were estimated as positive (90% [85%, 97%]), and nearly all were stronger than $SMD = 0.2$ (86% [80%, 93%]). Among only the MLR studies, the estimated average effect size was half as large (0.34 [0.27, 0.42]; $p<0.0001$) and with less estimated heterogeneity ($\widehat{\tau} = 0.11$). Despite the much smaller mean estimate in the MLR compared to the MA, we estimated that nearly all of the population effects were positive (100% [90%, 100%]) and that a large majority were stronger than $SMD = 0.2$ (89% [77%, 100%]), similar to the MA. This occurred because effects in the MLR were much more concentrated around their average than effects in the MA. Figures visualizing the population effects for each study, the densities of the distribution of population effects and pooled point estimates for each categorical candidate moderator are available in the project GitHub repository.

Statistical measure	Unadjusted model	Moderated model
$\widehat{\mu}$ in MA	0.64 [0.37, 0.91]	0.58 [0.29, 0.88]
$\widehat{\mu}$ in MLR	0.34 [0.27, 0.42]	0.14 [-0.07, 0.34]
$\widehat{\mu}$ discrepancy	0.29 [0.03, 0.56]	0.45 [0.02, 0.88]
% effects > 0 in MA	90 [85, 97]	98 [91, 100]
% effects > 0 in MLR	100 [90, 100]	100
% effects > 0 discrepancy	-10 [-15, -2]	-2 [-9, 0]
% effects > 0.2 in MA	86 [80, 93]	84 [81, 85]
% effects > 0.2 in MLR	89 [77, 100]	0
% effects > 0.2 discrepancy	-3 [-19, 12]	84 [-16, 87]

Table 2: *$\widehat{\mu}$: Average effect size (SMD), as estimated in a meta-regression model containing both sources (original MA and MLR). % effects > 0: Estimated percentage of positive population effects, as estimated in a meta-analysis or meta-regression model containing one source. % effects > 0.2: Estimated percentage of population effects stronger than $SMD = 0.2$. Discrepancies are calculated by subtracting between each statistical measure in the MLR from that in the MA, such that positive discrepancies indicate larger effect sizes in MA. Bracketed values are 95% confidence intervals, which are model-based for the $\widehat{\mu}$ measures (Hedges et al., 2010) and for differences in $\widehat{\mu}$ between sources and are bootstrapped for the percentage measures and for all cross-model comparisons (Mathur & VanderWeele, 2020a, 2021). Confidence intervals are omitted when they were not statistically estimable (i.e., for percentage estimates that were very close to 0% or 100%).*

In the **unadjusted model** that combined the two sources without any additional moderators, the estimated average effect sizes in the MA and in the MLR, respectively, were $SMD = 0.64$ (95% CI: [0.37, 0.91]) and 0.34 (95% CI: [0.27, 0.42]) (Table 2).[a] Thus, effect sizes in the MA were larger by on average 0.29 (95% CI: [0.03, 0.56]) units on the SMD scale. There was considerable residual heterogeneity (estimated standard deviation of population effects $\widehat{\tau}_{\text{unadjusted}} = 0.32$).

The **moderated model** converged when we included three moderators besides source: infant age, test language, and method (Table 3). In the moderated model, the estimated average effect size in the MA and in the MLR when setting the moderators to their average value (in the case of the continuous moderator infant age) or their most common value (in the case of the two categorical moderators, method and test language) in the MA was, respectively, 0.58 [0.29, 0.88] and 0.14 [−0.07, 0.34]. Thus, effect sizes in the MA were larger by, on average, 0.45 [0.02, 0.88] SMD units when controlling for these three moderators. This discrepancy was, if anything, larger than that seen in the unadjusted model, and the residual heterogeneity appeared essentially unchanged ($\widehat{\tau}_{\text{mod}} = 0.29$).

Finally, we also conducted the exploratory **interaction model** in which we included the two-way interaction between source (MA vs. MLR) and each of the three moderator variables

[a] These estimates differed negligibly from those obtained by fitting separate models to the MA and MLR studies. Separate models are not exactly equivalent to meta-regression because, for example, separate models involve separate heterogeneity estimates whereas meta-regression has a single, average heterogeneity estimate. The heterogeneity estimate in turn slightly affects estimates' relative weights in the model.

Moderator	Est	CI	p-value
Intercept	0.14	[-0.07, 0.34]	0.19
Source: Meta-Analysis	0.45	[0.02, 0.88]	0.04
Infant Age (months)	0.05	[0.02, 0.07]	< 0.001
Test Language: Non-native	-0.10	[-0.21, 0.01]	0.07
Test Language: Other	-0.46	[-2.40, 1.48]	0.40
Method: HPP	0.11	[-0.22, 0.43]	0.51
Method: Other	0.60	[-1.19, 2.38]	0.30

Table 3: *Meta-regression estimates (original MA) of moderation by various study design and participant characteristics. Intercept: estimated mean SMD when all listed moderators are set to 0 (for continuous moderators, the average value in the MA or, for categorical moderators, the most common value in the MA). The estimate of the categorical factor Meta-Analysis represents the change in SMD when this factor is true vs not. For infant age, the estimate represents the increase in effect size associated with a 1-month increase in mean participant age. For categorical moderators, estimates represent the increase compared to the reference level (Test Language: Native, and Method: Central Fixation, respectively). Bracketed values are 95% confidence intervals. p-values represent tests of moderators' coefficients themselves (vs. 0) in the meta-regression.*

(infant age, test language, and method). The results from this model are summarized in Table 4 and visualised in Figure 2. Note that we omit the publication bias analyses for the original MA here due to the many issues in reporting effect sizes identified in the dataset, which complicate any inferences about publication bias explaining the discrepancies between the original MA and the MLR.

Moderator	Est	CI	p-value
Intercept	0.32	[-0.05, 0.70]	0.08
Source (centered)	0.31	[-0.45, 1.06]	0.36
Age (months; centered)	0.07	[0.01, 0.12]	0.03
Test Language (Native vs. Other; centered)	0.12	[-0.20, 0.44]	0.27
Method (HPP vs. Other; centered)	-0.09	[-0.87, 0.69]	0.78
Source * Age	0.04	[-0.07, 0.16]	0.40
Source * Test Language	0.05	[-0.59, 0.69]	0.79
Source * Method	-0.65	[-2.20, 0.90]	0.34

Table 4: *Meta-regression estimates of the moderator interaction model (original MA). Intercept: estimated mean SMD when averaging across all (centered) moderators. Age (in months) is mean-centered. Test Language (Native vs. Other) and Method (HPP vs. Other) are treated as binary variables and centered. Bracketed values are 95% confidence intervals.*

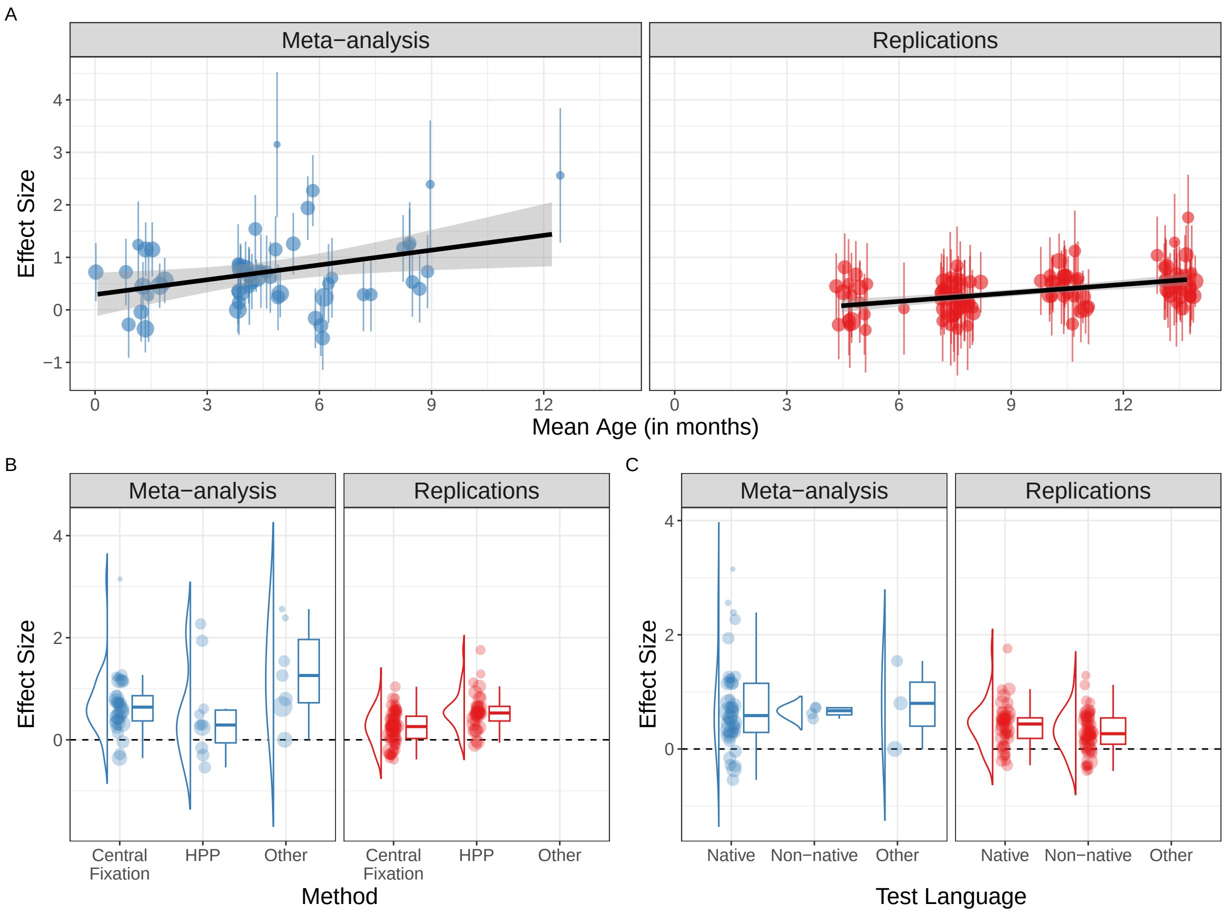

Figure 2: *Overview of the distribution of effect sizes in the meta-analysis (original MA) and replications (MLR) for three key moderators: infant age (A), method (B), and test language (C). In (A), the black line represents a linear fit through the effect sizes for each source and error bars for individual estimates are 95% confidence intervals.*

5.2. Results from the **revised** Dunst et al. (2012) meta-analysis

After amending the original Dunst et al. (2012) MA in light of several concerns — but still retaining only the papers included in the Dunst et al. (2012) MA —, the overall effect size in the revised Dunst et al. (2012) MA was $SMD = 0.50$ [0.27, 0.73] and the MA continued to contain substantial heterogeneity (estimated standard deviation of population effects $\widehat{\tau} = 0.32$). The meta-analytic effect size in the revised MA thus became more comparable to that of the MLR, with the difference between the two estimated as $SMD = 0.14$ $[-0.08, 0.36]$ ($p = 0.21$). **Figure 3** provides an overview over the population effects for studies in both studies, while **Figure 4** shows the estimated densities for both the marginal and conditional population effects. Note in both plots the greater heterogeneity exhibited by the revised MA compared to MLR. As in the results in the main text, we also visualize the pooled point estimates for the subset of studies in the revised MA and in the MLR for each categorical candidate moderator (**Figure 5**).

In the **unadjusted model** that combined the two sources without any additional

moderators, effect sizes in the revised MA were larger by on average 0.14 (95% CI: [−0.08, 0.36]) units on the SMD scale (Table 5). There was considerable residual heterogeneity (estimated standard deviation of population effects $\widehat{\tau}_{\text{unadjusted}} = 0.27$). Despite the slightly smaller mean estimate in the MLR compared to the MA, we again estimated that nearly all of the population effects for the MLR and the MA were positive and that a large majority were stronger than $SMD = 0.2$.

**Statistical measure**	**Unadjusted model**	**Moderated model**
$\widehat{\mu}$ in MA	0.48 [0.25, 0.71]	0.49 [0.2, 0.77]
$\widehat{\mu}$ in MLR	0.34 [0.27, 0.42]	0.2 [0.04, 0.37]
$\widehat{\mu}$ discrepancy	0.14 [-0.08, 0.36]	0.28 [-0.06, 0.62]
% effects $>$ 0 in MA	87 [87, 92]	98 [97, 99]
% effects $>$ 0 in MLR	100 [96, 100]	100
% effects $>$ 0 discrepancy	-13 [-14, -8]	-2 [-3, -1]
% effects $>$ 0.2 in MA	78 [76, 88]	93 [91, 96]
% effects $>$ 0.2 in MLR	89 [78, 100]	0
% effects $>$ 0.2 discrepancy	-11 [-25, 12]	93 [91, 96]

Table 5: *$\widehat{\mu}$: Average effect size (SMD), as estimated in a meta-regression model containing both sources (revised MA and MLR). % effects $>$ 0: Estimated percentage of positive population effects, as estimated in a meta-analysis or meta-regression model containing one source. % effects $>$ 0.2: Estimated percentage of population effects stronger than $SMD = 0.2$. Discrepancies are calculated by subtracting between each statistical measure in the MLR from that in the MA, such that positive discrepancies indicate larger effect sizes in MA. Bracketed values are 95% confidence intervals, which are model-based for the $\widehat{\mu}$ measures (Hedges et al., 2010) and for differences in $\widehat{\mu}$ between sources and are bootstrapped for the percentage measures and for all cross-model comparisons (Mathur & VanderWeele, 2020a, 2021). Confidence intervals are omitted when they were not statistically estimable (i.e., for percentage estimates that were very close to 0% or 100%).*

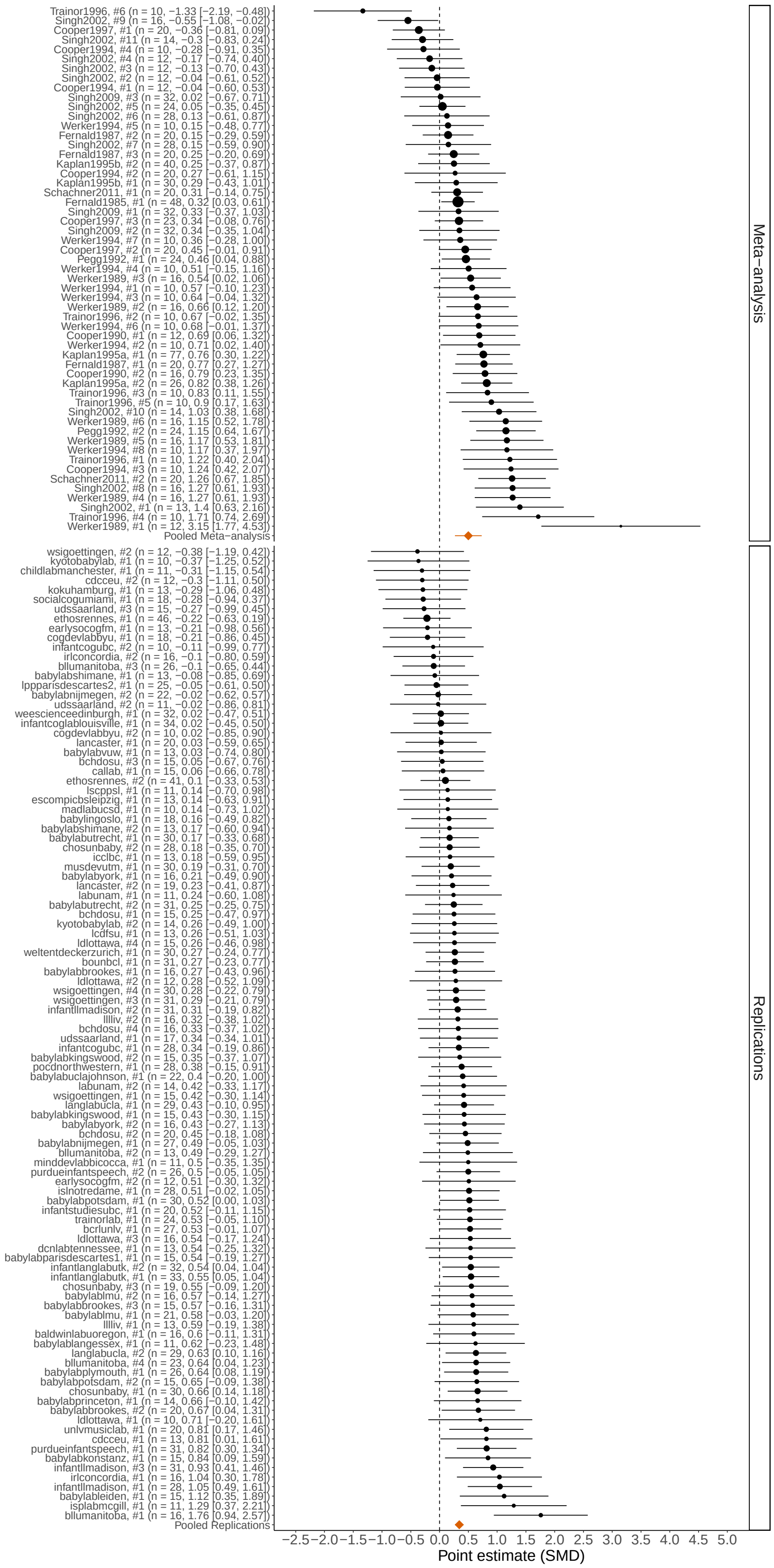

Figure 3: *Forest plot of studies' point estimates and 95% confidence intervals in the revised MA (top panel) and MLR (bottom panel). Orange diamond: pooled estimates within each source. Dashed vertical line: null.*

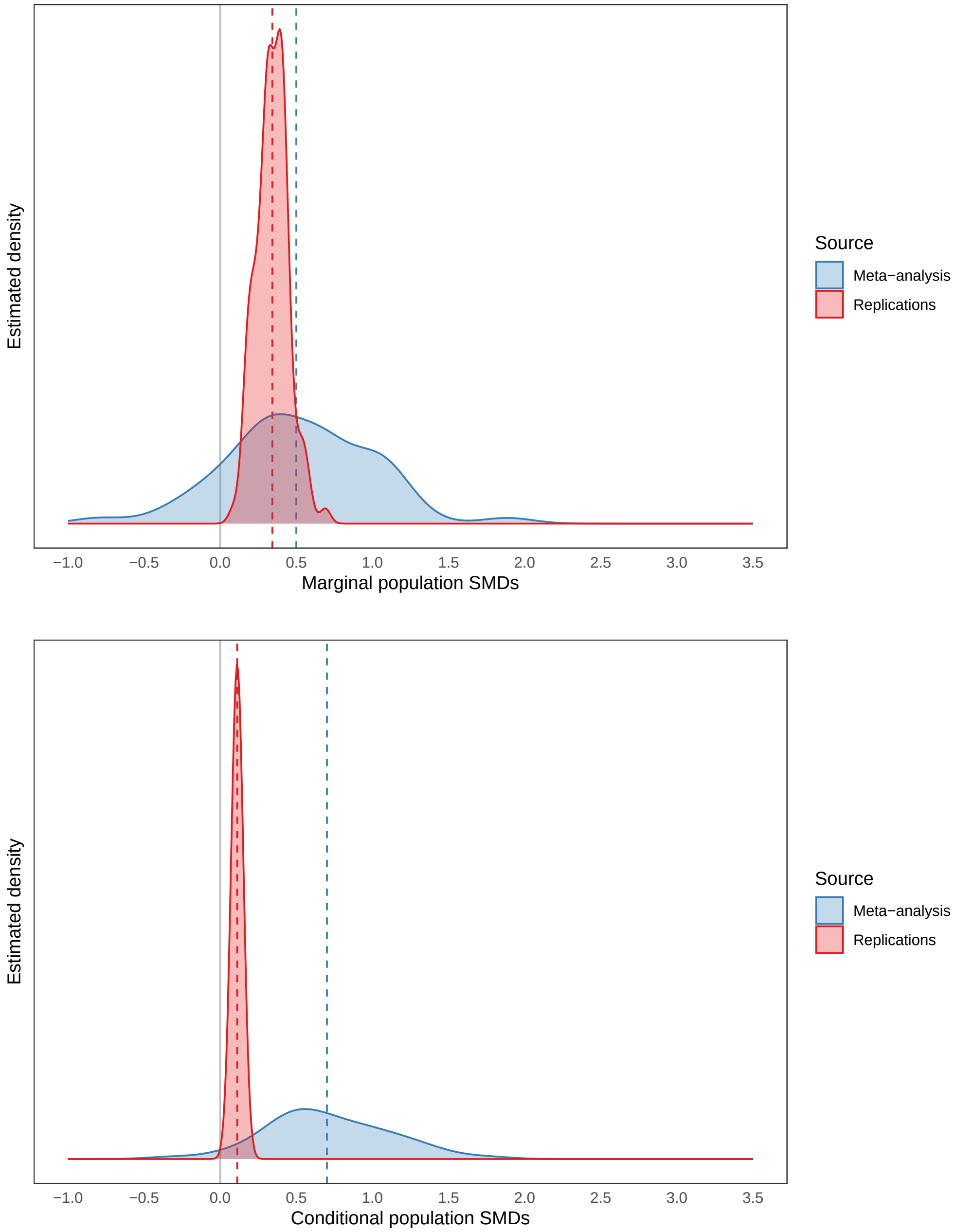

Figure 4: *Estimated densities of population effects in the revised MA (red) and in the MLR (gray). Top panel: Marginal population effects (i.e., not conditional on moderators). Bottom panel: Conditional population effects (i.e., conditional on the mean age and most common test language and method in the MA.) Vertical dashed lines: mean estimates from each source. Vertical gray line: null.*

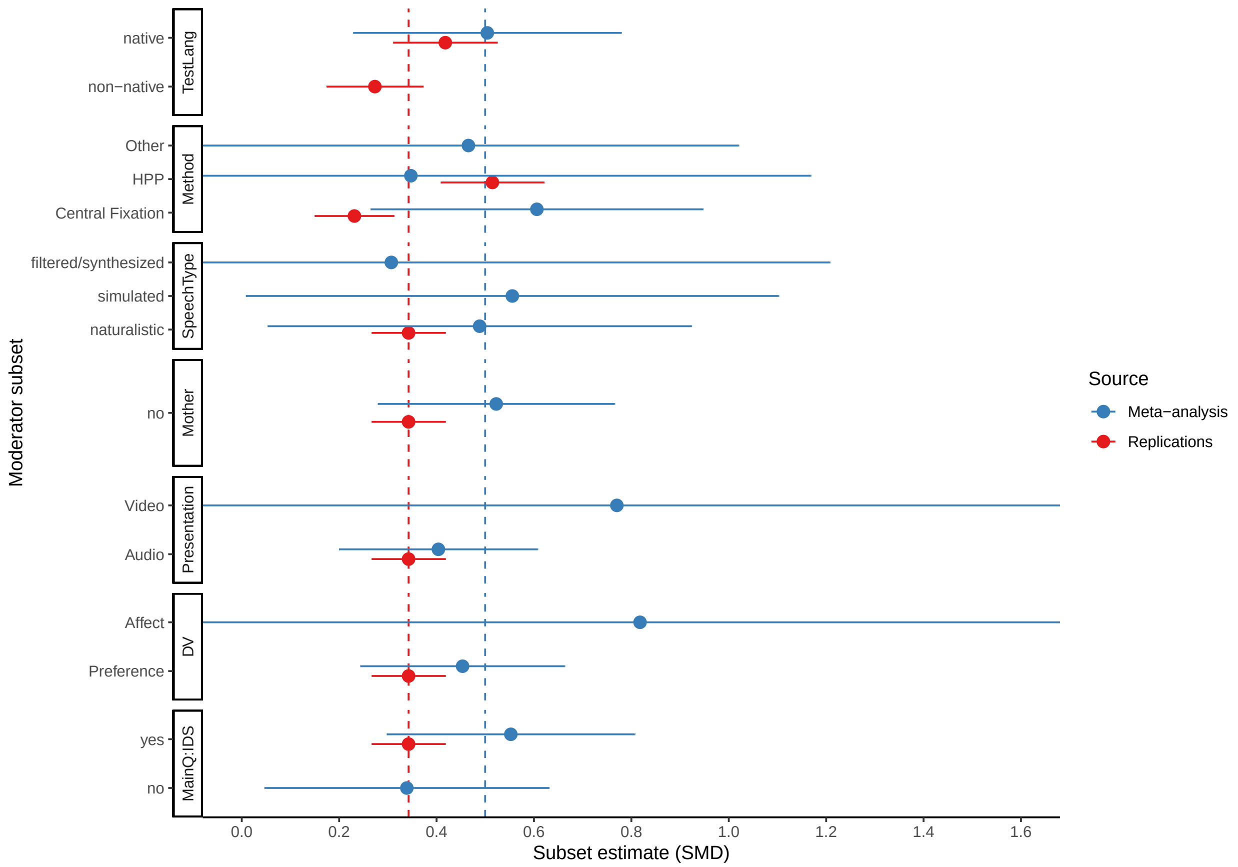

Figure 5: *Forest plot showing, for each categorical candidate moderator, the pooled point estimates for the subset of studies in the revised MA and in the MLR, respectively, with a given level of the moderator. Error bars are 95% confidence intervals. Error bars for many estimates are wide due to a limited number of observations at certain levels of a given moderator variable. Dashed vertical lines are unadjusted estimates in all MA studies and in all MLR studies.*

The **moderated model** converged when we included the same three moderators as before (besides source): infant age, test language, and method. **Table 6** summarizes the estimates of the meta-regression for the remaining moderators. The estimated average effect size in the revised MA and in the MLR when setting the moderators to their average values in the revised MA was, respectively, 0.49 [0.20, 0.77] and 0.20 [0.04, 0.37]. Thus, effect sizes in the MA were larger by, on average, 0.28 [−0.06, 0.62] SMD units when controlling for these three moderators ($p = 0.09$). This discrepancy was, again, larger than that seen in the unadjusted model, and the residual heterogeneity appeared essentially unchanged ($\widehat{\tau}_{\text{mod}} = 0.28$).

As in the main analysis, we additionally conducted the exploratory **interaction model** in which we included the two-way interaction between source (MA vs. MLR) and each of the three moderator variables (infant age, test language, and method). The results from this model are summarized in Table 7 and visualised in Figure 6.

Moderator	Est	CI	p-value
Intercept	0.2	[0.04, 0.37]	0.016
Source: Meta-Analysis	0.28	[-0.06, 0.62]	0.095
Age (months)	0.04	[0.02, 0.07]	0.001
Test Language: Non-native	-0.10	[-0.22, 0.02]	0.105
Test Language: Artificial	-0.15	[-2.42, 2.12]	0.558
Method: HPP	0.03	[-0.21, 0.26]	0.819
Method: Other	0.08	[-1.75, 1.91]	0.772

Table 6: *Meta-regression estimates (revised MA) of moderation by various study design and participant characteristics. Intercept: estimated mean SMD when all listed moderators are set to 0 (for continuous moderators, the average value in the MA or, for categorical moderators, the most common value in the MA). The estimate of the categorical factor Meta-Analysis represents the change in SMD when this factor is true vs not. For mean age, the estimate represents the increase in effect size associated with a 1-month increase in mean participant age. For categorical moderators, estimates represent the increase compared to the reference level (Test Language: Native, and Method: Central Fixation, respectively). Bracketed values are 95% confidence intervals. p-values represent tests of moderators' coefficients themselves (vs. 0) in the meta-regression.*

Moderator	Est	CI	p-value
Intercept	0.26	[0.13, 0.38]	0.003
Source (centered)	0.16	[-0.09, 0.41]	0.171
Age (months; centered)	0.05	[-0.00, 0.11]	0.051
Test Language (Native vs. Other; centered)	0.15	[-0.14, 0.43]	0.174
Method (HPP vs. Other; centered)	-0.1	[-0.37, 0.17]	0.401
Source * Age	0.02	[-0.09, 0.13]	0.669
Source * Test Language	0.11	[-0.46, 0.68]	0.549
Source * Method	-0.67	[-1.22, -0.13]	0.0217

Table 7: *Meta-regression estimates of the moderator interaction model (revised MA). Intercept: estimated mean SMD when averaging across all (centered) moderators. Age (in months) is mean-centered. Test Language (Native vs. Other) and Method (HPP vs. Other) are treated as binary variables and centered. Bracketed values are 95% confidence intervals.*

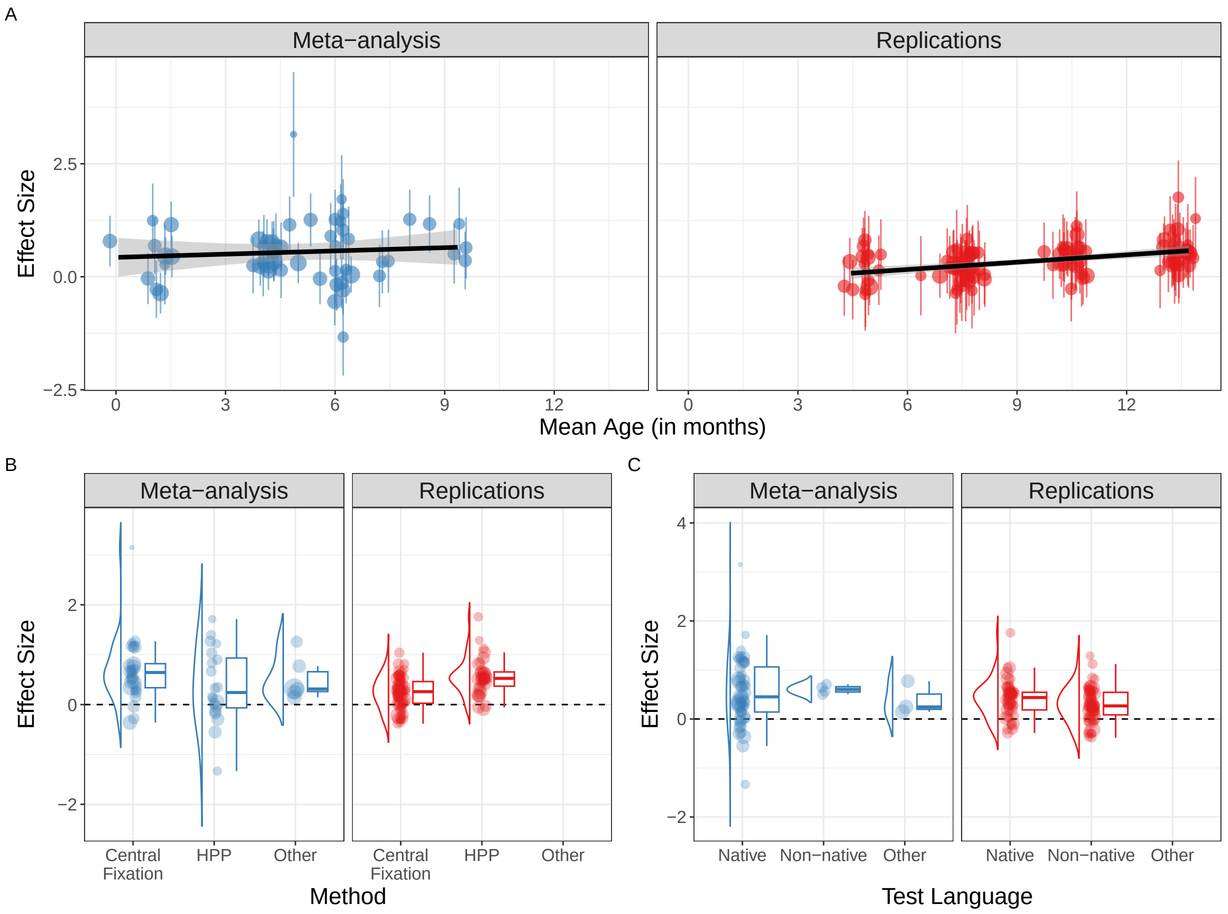

Figure 6: *Overview of the distribution of effect sizes in the meta-analysis (revised MA) and replications (MLR) for three key moderators: infant age (A), method (B), and test language (C). In (A), the black line represents a linear fit through the effect sizes for each source and error bars for individual estimates are 95% confidence intervals.*

Finally, we also considered publication bias as a possible source of differences between the revised MA and MLR. The revised MA contained 25 affirmative and 30 nonaffirmative studies (vs 28 and 23, respectively in the original dataset). We began by implementing a correction for publication bias, estimating the level of affirmative selection from the MA itself. The average effect size in the MA after correction was $SMD = 0.41$ [0.18, 0.64]; $p = 5.0000 \cdot 10^{-4}$ (Vevea & Hedges, 1995), which was in this case indeed smaller than the uncorrected estimate of $SMD = 0.50$. Next we applied sensitivity analyses for publication bias, considering what the true effect size would be under several different scenarios. Under hypothetical worst-case publication bias (i.e., if "statistically significant" positive results were infinitely more likely to be published than "nonsignificant" or negative results), the MA mean would decrease to 0.12 [−0.15, 0.39], which was in fact less than the estimate in the MLRs. Under "typical" publication bias in this field (favoring affirmative results by 4.7-fold), the MA average would decrease to 0.25 [−0.01, 0.50]. In both cases these estimates decreased sharply, were numerically smaller than MLR, and included zero in the 95% CI. We took this finding as suggesting that publication bias could therefore explain the discrepancy between the MA

and MLR, which further motivated us to expand the MA to include papers and unpublished datasets beyond those included in the original Dunst et al. (2012) MA.

5.3. Overview of Results across Meta-Analytic Datasets

To obtain an overview of how the findings differ across each of the three meta-analytic datasets (i.e., the original, the corrected and the community-augmented), Table 8 shows how key estimates from each of the models change with each meta-analytic dataset.

	Original Dunst	Revised Dunst	Community-Augmented
Total Studies	16	14	30
Total Estimates	51	55	112
MA Estimate Alone	0.67 [0.38, 0.95]	0.50 [0.27, 0.73]	0.35 [0.22, 0.47]
Unadjusted Estimate	0.64 [0.37, 0.91]	0.48 [0.25, 0.71]	0.34 [0.22, 0.46]
Heterogeneity Estimate	0.32	0.27	0.27
Moderated Estimate	0.58 [0.29, 0.88]	0.49 [0.20, 0.77]	0.32 [0.16, 0.47]
Significant Moderators (Moderated Model)	Source, Age	Age	None
Significant Interactions (Interaction Model)	None	Source*Method	Source*Method & Source*Age

Table 8: *This table provides an overview of key differences in properties and effects across each meta-analytic dataset: 1) the original dataset from Dunst et al. (2012), 2) the revised dataset from Dunst et al. (2012) with a variety of revisions and corrections (see Section 2 for details), and 3) the community-augmented dataset used for the analyses in the main manuscript that augments the revised Dunst et al. (2012) dataset based on an updated literature search. MA Estimate Alone refers to the meta-analytic estimate when the meta-analtic data is modeled separately from the MLR data. Unadjusted Estimate refers to the intercept estimate from the unadjusted model. Heterogeneity Estimate is the estimated standard deviation of population effects in the unadjusted model. Moderated Estimate refers to the intercept estimate from the model including key moderators. Significant moderators are from the preregistered moderated model including data source, experimental task, age, and native language as predictors (no interactions between moderators). Significant interactions are from the exploratory interaction model testing whether the effect of experimental task, age, and native language between data source, i.e. the (respective) MA and the MLR.*

5.4. Additional analyses with the community-augmented meta-analysis

5.4.1 Overview over included studies and effect sizes in the MA

Table 9: *Overview of the effect size estimates for each individual sample included in the community-augmented MA. Bracketed values are 95% confidence intervals.*

Study	N	Mean Age (mos.)	Test Language	Method	Estimate	CI
Cooper & Aslin (1990)	12	1.1	Native	Central fixation	0.69	[0.06, 1.32]
Cooper & Aslin (1990)	16	0.1	Native	Central fixation	0.79	[0.23, 1.35]

Cooper & Aslin (1994)	12	1.1	Native	Central fixation	-0.04	[-0.60, 0.53]
Cooper & Aslin (1994)	20	1.1	Native	Central fixation	0.27	[-0.61, 1.15]
Cooper & Aslin (1994)	10	1.1	Native	Central fixation	1.24	[0.42, 2.07]
Cooper & Aslin (1994)	10	1.1	Native	Central fixation	-0.28	[-0.91, 0.35]
Cooper et al. (1997)	20	1.3	Native	Central fixation	-0.36	[-0.81, 0.09]
Cooper et al. (1997)	20	1.4	Native	Central fixation	0.45	[-0.01, 0.91]
Cooper et al. (1997)	23	4.1	Native	Central fixation	0.34	[-0.08, 0.76]
Corbeil et al. (2016)	20	8.5	Non-Native	Central fixation	0.10	[-0.78, 0.98]
Droucker et al. (2013)	22	6.0	Native	Central fixation	0.29	[-0.14, 0.71]
Droucker et al. (2013)	22	8.0	Native	Central fixation	0.29	[-0.14, 0.72]
Droucker et al. (2013)	22	12.0	Native	Central fixation	0.24	[-0.18, 0.67]
Droucker et al. (2013)	22	18.0	Native	Central fixation	0.52	[0.08, 0.97]
Droucker et al. (2013)	14	6.0	Native	Central fixation	-0.01	[-0.53, 0.52]
Droucker et al. (2013)	14	8.0	Native	Central fixation	0.11	[-0.42, 0.64]
Droucker et al. (2013)	14	12.0	Native	Central fixation	0.29	[-0.25, 0.82]
Droucker et al. (2013)	14	18.0	Native	Central fixation	0.70	[0.11, 1.28]
Fernald & Kuhl (1987)	20	4.1	Artificial	Other	0.77	[0.27, 1.27]
Fernald & Kuhl (1987)	20	4.0	Artificial	Other	0.15	[-0.29, 0.59]
Fernald & Kuhl (1987)	20	4.1	Artificial	Other	0.25	[-0.20, 0.69]
Fernald (1985)	48	4.0	Native	Other	0.32	[0.03, 0.61]
Hayashi et al. (2001)	24	5.5	Native	HPP	0.77	[0.32, 1.23]
Hayashi et al. (2001)	31	8.5	Native	HPP	0.09	[-0.26, 0.44]
Hayashi et al. (2001)	34	12.4	Native	HPP	1.38	[0.91, 1.85]
Inoue et al. (2011)	17	8.0	Native	HPP	0.48	[-0.03, 0.98]
Inoue et al. (2011)	17	8.4	Native	HPP	0.12	[-0.35, 0.60]
Kaplan et al. (1995a)	77	4.1	Native	Central fixation	0.76	[0.30, 1.22]
Kaplan et al. (1995a)	26	4.1	Native	Central fixation	0.82	[0.38, 1.26]
Kaplan et al. (1995b)	30	4.1	Native	Central fixation	0.29	[-0.43, 1.01]
Kaplan et al. (1995b)	40	4.0	Native	Central fixation	0.25	[-0.37, 0.87]
Kaplan et al. 2018	45	9.0	Native	Central fixation	0.31	[0.02, 0.61]
Kaplan et al. 2018	21	8.2	Native	Central fixation	-0.12	[-0.55, 0.31]
Kim & Johnson (2014)	42	5.2	Native	Central fixation	0.39	[0.08, 0.71]
Kim & Johnson (2014)	42	5.2	Native	Central fixation	-0.12	[-0.43, 0.18]
Kim & Johnson (2014)	33	3.1	Native	Central fixation	0.43	[0.08, 0.79]
Kim & Johnson (2014)	33	3.1	Native	Central fixation	0.24	[-0.11, 0.58]
McCartney (1997)	24	4.3	Native	Central fixation	-0.14	[-0.54, 0.26]
McFayden et al. (2020)	10	14.5	Native	Central fixation	0.63	[-0.05, 1.31]
McFayden et al. (2020)	10	14.5	Native	Central fixation	0.62	[-0.06, 1.30]
Newman & Hussain (2006)	30	4.3	Native	HPP	0.17	[-0.19, 0.53]
Newman & Hussain (2006)	30	4.3	Native	HPP	0.43	[0.05, 0.80]
Newman & Hussain (2006)	30	8.9	Native	HPP	0.06	[-0.30, 0.41]
Newman & Hussain (2006)	30	8.9	Native	HPP	-0.16	[-0.52, 0.20]
Newman & Hussain (2006)	30	13.3	Native	HPP	-0.04	[-0.40, 0.32]
Newman & Hussain (2006)	30	13.3	Native	HPP	-0.23	[-0.59, 0.13]
Newman (unpublished)	24	4.9	Native	HPP	-0.01	[-0.41, 0.39]
Newman (unpublished)	24	4.9	Native	HPP	0.11	[-0.29, 0.52]
Newman (unpublished)	15	4.8	Native	HPP	-0.08	[-0.59, 0.42]
Newman (unpublished)	15	4.8	Native	HPP	0.19	[-0.32, 0.70]
Ostroff (1998)	20	10.4	Non-Native	Central fixation	0.55	[0.08, 1.02]
Pegg et al. (1992)	24	1.6	Native	Central fixation	0.46	[0.04, 0.88]
Pegg et al. (1992)	24	1.6	Native	Central fixation	1.15	[0.64, 1.67]
Robertson et al. (2013)	9	19.1	Native	Central fixation	0.95	[0.16, 1.74]
Robertson et al. (2013)	9	7.8	Native	Central fixation	0.38	[-0.30, 1.05]
Robertson et al. (2013)	9	18.6	Native	Central fixation	0.04	[-0.61, 0.70]
Schachner & Hannon (2011)	20	5.2	Native	Other	0.31	[-0.14, 0.75]
Schachner & Hannon (2011)	20	5.2	Native	Other	1.26	[0.67, 1.85]
Segal & Newman (2015)	36	12.0	Native	HPP	0.25	[-0.08, 0.58]
Segal & Newman (2015)	24	16.0	Native	HPP	0.35	[-0.06, 0.76]
Segal & Newman (2015)	36	12.0	Native	HPP	0.07	[-0.26, 0.40]
Segal & Newman (2015)	24	16.0	Native	HPP	0.01	[-0.39, 0.41]
Singh et al. (2002)	13	6.1	Native	HPP	1.40	[0.63, 2.16]
Singh et al. (2002)	12	5.9	Native	HPP	-0.04	[-0.61, 0.52]
Singh et al. (2002)	12	5.9	Native	HPP	-0.13	[-0.70, 0.43]
Singh et al. (2002)	12	5.9	Native	HPP	-0.17	[-0.74, 0.40]
Singh et al. (2002)	24	6.2	Native	HPP	0.05	[-0.35, 0.45]
Singh et al. (2002)	28	6.1	Native	HPP	0.13	[-0.61, 0.87]
Singh et al. (2002)	28	6.1	Native	HPP	0.15	[-0.59, 0.90]
Singh et al. (2002)	16	6.0	Native	HPP	1.27	[0.61, 1.93]

Singh et al. (2002)	16	6.0	Native	HPP	-0.55	[-1.08, -0.02]
Singh et al. (2002)	14	6.1	Native	HPP	1.03	[0.38, 1.68]
Singh et al. (2002)	14	6.1	Native	HPP	-0.30	[-0.83, 0.24]
Singh et al. (2009)	32	7.4	Native	HPP	0.33	[-0.37, 1.03]
Singh et al. (2009)	32	7.4	Native	HPP	0.34	[-0.35, 1.04]
Singh et al. (2009)	32	7.4	Native	HPP	0.02	[-0.67, 0.71]
Trainor et al. (1996)	10	6.1	Native	HPP	1.22	[0.40, 2.04]
Trainor et al. (1996)	10	6.1	Native	HPP	0.67	[-0.02, 1.35]
Trainor et al. (1996)	10	6.1	Native	HPP	0.83	[0.11, 1.55]
Trainor et al. (1996)	10	6.1	Native	HPP	1.71	[0.74, 2.69]
Trainor et al. (1996)	10	6.1	Native	HPP	0.90	[0.17, 1.63]
Trainor et al. (1996)	10	6.1	Native	HPP	-1.33	[-2.19, -0.48]
Wang et al. (2017)	12	27.2	Native	Central fixation	0.87	[0.21, 1.54]
Wang et al. (2017)	22	11.7	Native	Central fixation	0.45	[0.01, 0.89]
Wang et al. (2017)	12	27.6	Native	Central fixation	-0.02	[-0.59, 0.54]
Wang et al. (2017)	12	27.2	Native	Central fixation	0.66	[0.04, 1.28]
Wang et al. (2017)	22	11.7	Native	Central fixation	0.42	[-0.01, 0.86]
Wang et al. (2017)	12	27.6	Native	Central fixation	0.62	[0.00, 1.24]
Wang et al. (2018)	9	17.6	Native	Central fixation	0.09	[-0.56, 0.75]
Wang et al. (2018)	9	17.5	Native	Central fixation	0.26	[-0.40, 0.93]
Wang et al. (2018)	10	9.9	Native	Central fixation	0.84	[0.12, 1.56]
Wang et al. (2018)	14	9.1	Native	Central fixation	0.57	[0.01, 1.14]
Wang et al. (2018)	9	17.6	Native	Central fixation	-0.06	[-0.72, 0.59]
Wang et al. (2018)	9	17.5	Native	Central fixation	0.34	[-0.33, 1.01]
Wang et al. (2018)	10	9.9	Native	Central fixation	-0.83	[-1.55, -0.11]
Wang et al. (2018)	14	9.1	Native	Central fixation	-0.16	[-0.69, 0.37]
Ward & Cooper (1999)	40	4.3	Native	Central fixation	-0.68	[-1.32, -0.04]
Ward & Cooper (1999)	40	4.3	Native	Central fixation	-0.63	[-1.26, 0.01]
Werker & McLeod (1989)	12	5.1	Native	Central fixation	3.15	[1.77, 4.53]
Werker & McLeod (1989)	16	4.4	Native	Central fixation	0.66	[0.12, 1.20]
Werker & McLeod (1989)	16	4.4	Native	Central fixation	0.54	[0.02, 1.06]
Werker & McLeod (1989)	16	8.3	Native	Central fixation	1.27	[0.61, 1.93]
Werker & McLeod (1989)	16	8.3	Native	Central fixation	1.17	[0.53, 1.81]
Werker & McLeod (1989)	16	4.6	Native	Central fixation	1.15	[0.52, 1.78]
Werker et al. (1994)	10	4.3	Non-Native	Central fixation	0.57	[-0.10, 1.23]
Werker et al. (1994)	10	4.3	Non-Native	Central fixation	0.71	[0.02, 1.40]
Werker et al. (1994)	10	9.4	Non-Native	Central fixation	0.64	[-0.04, 1.32]
Werker et al. (1994)	10	9.4	Non-Native	Central fixation	0.51	[-0.15, 1.16]
Werker et al. (1994)	10	4.3	Native	Central fixation	0.15	[-0.48, 0.77]
Werker et al. (1994)	10	4.3	Native	Central fixation	0.68	[-0.01, 1.37]
Werker et al. (1994)	10	9.4	Native	Central fixation	0.36	[-0.28, 1.00]
Werker et al. (1994)	10	9.4	Native	Central fixation	1.17	[0.37, 1.97]

5.4.2 Overview over included studies and effect sizes in the multi-lab replication

Table 10: *Overview of the effect size estimates for each individual sample included in the MLR. Bracketed values are 95% confidence intervals.*

Lab ID	N	Mean Age (mos.)	Test Language	Method	Estimate	CI
babylabbrookes	16	13.6	Non-Native	Central fixation	0.27	[-0.43, 0.96]
babylabbrookes	20	4.5	Non-Native	Central fixation	0.67	[0.04, 1.31]
babylabbrookes	15	7.6	Non-Native	Central fixation	0.57	[-0.16, 1.31]
babylabkingswood	15	13.4	Non-Native	HPP	0.43	[-0.30, 1.15]
babylabkingswood	15	7.3	Non-Native	HPP	0.35	[-0.37, 1.07]
babylabkonstanz	15	7.7	Non-Native	HPP	0.84	[0.09, 1.59]
babylablangessex	11	7.3	Non-Native	Central fixation	0.62	[-0.23, 1.48]
babylableiden	15	10.5	Non-Native	HPP	1.12	[0.35, 1.89]
babylablmu	21	13.6	Non-Native	Central fixation	0.58	[-0.03, 1.20]
babylablmu	16	10.6	Non-Native	Central fixation	0.57	[-0.14, 1.27]
babylabnijmegen	27	7.7	Non-Native	HPP	0.49	[-0.05, 1.03]
babylabnijmegen	22	10.8	Non-Native	HPP	-0.02	[-0.62, 0.57]
babylabparisdescartes1	15	13.3	Non-Native	HPP	0.54	[-0.19, 1.27]
babylabplymouth	26	10.5	Non-Native	HPP	0.64	[0.08, 1.19]
babylabpotsdam	30	10.1	Non-Native	HPP	0.52	[0.00, 1.03]

babylabpotsdam	15	10.1	Non-Native	Central fixation	0.65	[-0.09, 1.38]
babylabprinceton	14	13.6	Native	HPP	0.66	[-0.10, 1.42]
babylabshimane	13	4.9	Non-Native	Central fixation	-0.08	[-0.85, 0.69]
babylabshimane	13	7.6	Non-Native	Central fixation	0.17	[-0.60, 0.94]
babylabuclajohnson	22	13.3	Native	Central fixation	0.40	[-0.20, 1.00]
babylabutrecht	30	7.4	Non-Native	HPP	0.17	[-0.33, 0.68]
babylabutrecht	31	10.7	Non-Native	HPP	0.25	[-0.25, 0.75]
babylabvuw	13	7.5	Non-Native	Central fixation	0.03	[-0.74, 0.80]
babylabyork	16	7.2	Non-Native	Central fixation	0.21	[-0.49, 0.90]
babylabyork	16	10.4	Non-Native	Central fixation	0.43	[-0.27, 1.13]
babylingoslo	18	7.5	Non-Native	Central fixation	0.16	[-0.49, 0.82]
baldwinlabuoregon	16	10.5	Native	Central fixation	0.60	[-0.11, 1.31]
bchdosu	15	13.5	Native	Central fixation	0.25	[-0.47, 0.97]
bchdosu	20	4.6	Native	Central fixation	0.45	[-0.18, 1.08]
bchdosu	15	8.0	Native	Central fixation	0.05	[-0.67, 0.76]
bchdosu	16	10.6	Native	Central fixation	0.33	[-0.37, 1.02]
bcrlunlv	27	13.5	Native	Central fixation	0.53	[-0.01, 1.07]
bllumanitoba	16	13.6	Native	HPP	1.76	[0.94, 2.57]
bllumanitoba	13	5.4	Native	HPP	0.49	[-0.29, 1.27]
bllumanitoba	26	7.7	Native	HPP	-0.10	[-0.65, 0.44]
bllumanitoba	23	10.3	Native	HPP	0.64	[0.04, 1.23]
bounbcl	31	13.4	Non-Native	Central fixation	0.27	[-0.23, 0.77]
callab	15	11.0	Native	Central fixation	0.06	[-0.66, 0.78]
cdcceu	13	13.1	Non-Native	Central fixation	0.81	[0.01, 1.61]
cdcceu	12	4.8	Non-Native	Central fixation	-0.30	[-1.11, 0.50]
childlabmanchester	11	7.9	Non-Native	Central fixation	-0.31	[-1.15, 0.54]
chosunbaby	30	13.3	Non-Native	HPP	0.66	[0.14, 1.18]
chosunbaby	28	7.5	Non-Native	HPP	0.18	[-0.35, 0.70]
chosunbaby	19	9.7	Non-Native	HPP	0.55	[-0.09, 1.20]
cogdevlabbyu	18	4.5	Native	Central fixation	-0.21	[-0.86, 0.45]
cogdevlabbyu	10	6.4	Native	Central fixation	0.02	[-0.85, 0.90]
dcnlabtennessee	13	13.3	Native	Central fixation	0.54	[-0.25, 1.32]
earlysocogfm	13	7.4	Native	Central fixation	-0.21	[-0.98, 0.56]
earlysocogfm	12	10.4	Native	Central fixation	0.51	[-0.30, 1.32]
escompicbsleipzig	13	5.1	Non-Native	Central fixation	0.14	[-0.63, 0.91]
ethosrennes	46	4.8	Non-Native	Central fixation	-0.22	[-0.63, 0.19]
ethosrennes	41	7.7	Non-Native	Central fixation	0.10	[-0.33, 0.53]
icclbc	13	7.6	Native	Central fixation	0.18	[-0.59, 0.95]
infantcoglablouisville	34	10.7	Native	Central fixation	0.02	[-0.45, 0.50]
infantcogubc	28	4.6	Native	Central fixation	0.34	[-0.19, 0.86]
infantcogubc	10	7.4	Native	Central fixation	-0.11	[-0.99, 0.77]
infantlanglabutk	33	13.7	Native	HPP	0.55	[0.05, 1.04]
infantlanglabutk	32	7.4	Native	HPP	0.54	[0.04, 1.04]
infantllmadison	28	13.5	Native	HPP	1.05	[0.49, 1.61]
infantllmadison	31	7.3	Native	HPP	0.31	[-0.19, 0.82]
infantllmadison	31	10.4	Native	HPP	0.93	[0.41, 1.46]
infantstudiesubc	20	7.5	Native	HPP	0.52	[-0.11, 1.15]
irlconcordia	16	13.0	Native	Central fixation	1.04	[0.30, 1.78]
irlconcordia	16	7.2	Native	Central fixation	-0.10	[-0.80, 0.59]
islnotredame	28	13.5	Native	HPP	0.51	[-0.02, 1.05]
isplabmcgill	11	13.6	Non-Native	HPP	1.29	[0.37, 2.21]
kokuhamburg	13	7.4	Non-Native	Central fixation	-0.29	[-1.06, 0.48]
kyotobabylab	10	7.3	Non-Native	Central fixation	-0.37	[-1.25, 0.52]
kyotobabylab	14	9.8	Non-Native	Central fixation	0.26	[-0.49, 1.00]
labunam	11	13.3	Non-Native	Central fixation	0.24	[-0.60, 1.08]
labunam	14	7.3	Non-Native	Central fixation	0.42	[-0.33, 1.17]
lancaster	20	13.4	Non-Native	Central fixation	0.03	[-0.59, 0.65]
lancaster	19	7.4	Non-Native	Central fixation	0.23	[-0.41, 0.87]
langlabucla	29	5.1	Native	HPP	0.43	[-0.10, 0.95]
langlabucla	29	10.7	Native	HPP	0.63	[0.10, 1.16]
lcdfsu	13	10.5	Native	Central fixation	0.26	[-0.51, 1.03]
ldlottawa	10	13.6	Native	Central fixation	0.71	[-0.20, 1.61]
ldlottawa	12	4.8	Native	Central fixation	0.28	[-0.52, 1.09]
ldlottawa	16	7.9	Native	Central fixation	0.54	[-0.17, 1.24]
ldlottawa	15	10.4	Native	Central fixation	0.26	[-0.46, 0.98]
lllliv	13	13.1	Non-Native	Central fixation	0.59	[-0.19, 1.38]
lllliv	16	7.4	Non-Native	Central fixation	0.32	[-0.38, 1.02]
lppparisdescartes2	25	7.9	Non-Native	HPP	-0.05	[-0.61, 0.50]

lscppsl	11	13.2	Non-Native	Central fixation	0.14	[-0.70, 0.98]
madlabucsd	10	7.7	Native	Central fixation	0.14	[-0.73, 1.02]
minddevlabbicocca	11	4.8	Non-Native	Central fixation	0.50	[-0.35, 1.35]
musdevutm	30	7.4	Native	HPP	0.19	[-0.31, 0.70]
pocdnorthwestern	28	13.4	Native	Central fixation	0.38	[-0.15, 0.91]
purdueinfantspeech	31	12.9	Native	HPP	0.82	[0.30, 1.34]
purdueinfantspeech	26	10.3	Native	HPP	0.50	[-0.05, 1.05]
socialcogumiami	18	4.5	Native	Central fixation	-0.28	[-0.94, 0.37]
trainorlab	24	8.0	Native	HPP	0.53	[-0.05, 1.10]
udssaarland	17	13.4	Non-Native	Central fixation	0.34	[-0.34, 1.01]
udssaarland	11	7.8	Non-Native	Central fixation	-0.02	[-0.86, 0.81]
udssaarland	15	10.4	Non-Native	Central fixation	-0.27	[-0.99, 0.45]
unlvmusiclab	20	4.6	Native	Central fixation	0.81	[0.17, 1.46]
weescienceedinburgh	32	7.0	Non-Native	Central fixation	0.02	[-0.47, 0.51]
weltentdeckerzurich	30	13.6	Non-Native	Central fixation	0.27	[-0.24, 0.77]
wsigoettingen	15	13.5	Non-Native	Central fixation	0.42	[-0.30, 1.14]
wsigoettingen	12	4.8	Non-Native	Central fixation	-0.38	[-1.19, 0.42]
wsigoettingen	31	7.3	Non-Native	Central fixation	0.29	[-0.21, 0.79]
wsigoettingen	30	10.2	Non-Native	Central fixation	0.28	[-0.22, 0.79]

5.4.3 Estimated densities of population effects in the MA and MLR

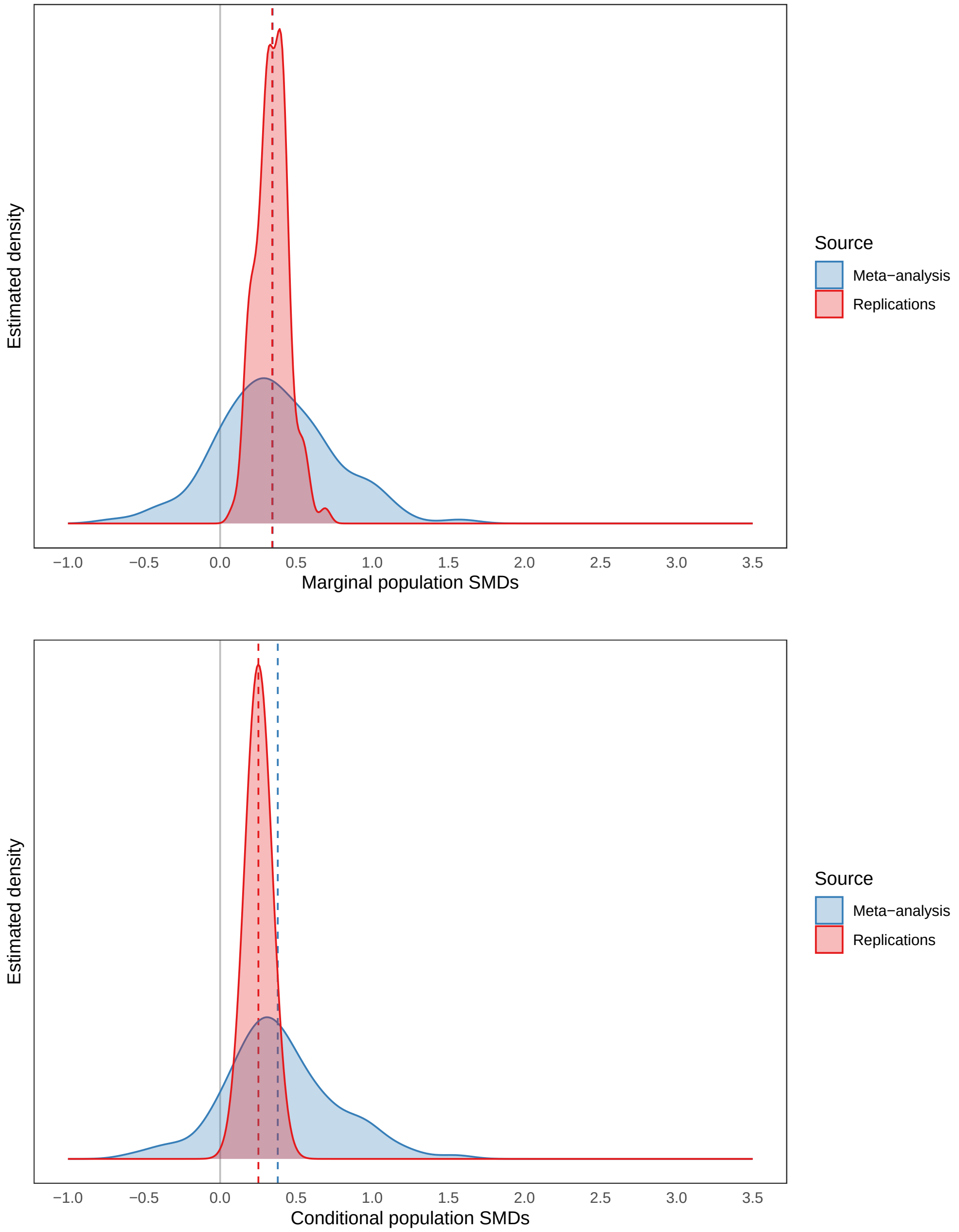

Figure 7: *Estimated densities of population effects in the community-augmented MA (red) and in the MLR (gray). Top panel: Unadjusted population effects (i.e., not conditional on moderators). Bottom panel: Conditional population effects (i.e., conditional on the mean infant age and most common test language and method in the MA.) Vertical dashed lines: mean estimates from each source. Note that in the top panel, the two estimates almost completely overlap. Vertical gray line: null.*

5.4.4 Sensitivity analysis: Within-subjects experiment design

A small number of studies in the MA used between-subjects rather than within-subjects designs. We anticipated that between-subjects designs may differ systematically from within-subjects designs because larger unintended variation between conditions in between-subjects designs is not necessarily countered by increasing the sample size in infant research, since testing is costly (Bergmann et al., 2018). We therefore repeated the analyses in Section 3.1 of the main manuscript after excluding from the MA the 12 estimates from between-subjects designs; doing so slightly increased the average effect size in the MA from 0.35 [0.22, 0.47] to 0.37 [0.24, 0.50]. The meta-regression results for both the unadjusted and moderated models corroborated the main results, suggesting that the use of between-subjects designs in some of the MA studies did not explain the discrepancies.

5.4.5 Additional analyses supporting publication bias methods

Visual diagnostics for assumptions. To assess for violations of the assumption that publication bias operates in favor of affirmative results (i.e., those with $p < 0.05$ and point estimates in the desired direction), we calculated and plotted one-tailed p-values from the meta-analysis studies (Figure 8). The much larger mass of one-tailed p-values below 0.025 (37.00% of all p-values) versus those above 0.975 (4.0% of p-values) suggested that any selection, if present, indeed was one-directional rather than two-directional.

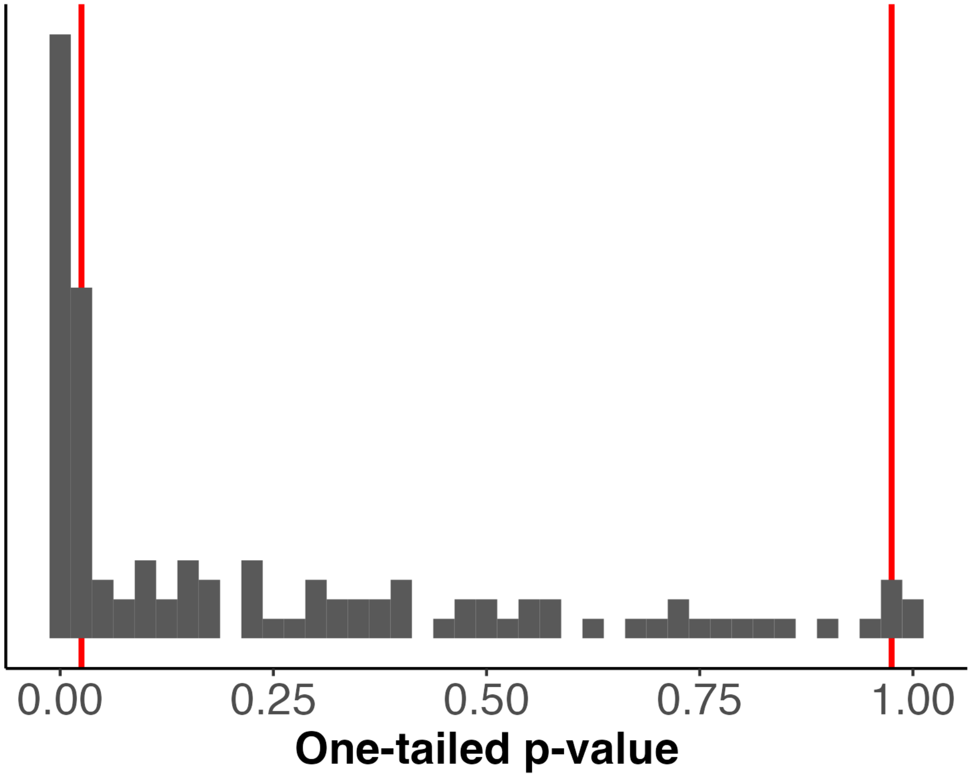

Figure 8: *One-tailed p-values from all studies in the meta-analysis. Red lines indicate the 0.025 and 0.975 thresholds, i.e., the thresholds at which the corresponding two-tailed p-value would be* < 0.05 *and in the desired direction and at which the two-tailed p-value would be* < 0.05 *but in the unanticipated direction.*

Paper-reported significance. Additionally, our main analyses defined statistical "significance" and affirmative status based on whether our calculated p-value was less than 0.05. Our calculated p-value sometimes differed from the p-value reported in the papers, likely due to differences in the statistical strategy used (e.g., the original study might have computed p-values using a one-tailed test) or (in some instances) due to a potential error in the original study. Of p-values that we calculated to be less than 0.05, authors reported 97% to be "significant"; and of p-values that we calculated to be greater than 0.05 (i.e., non-significant), authors in the original studies reported 29% to be "significant". We repeated the sensitivity analyses under the alternative assumption that publication bias favors results that studies' authors reported to be significant and positive (Mathur & VanderWeele, 2020b). These analyses yielded generally similar results, except that the estimate under hypothetical worst-case publication bias (0.04 [−0.11, 0.18]) was somewhat smaller than in main analyses (0.09 [−0.01, 0.18]).

5.4.6 Exploratory sensitivity analysis: Applying more stringent participant inclusion criteria in the multi-lab replication

Exploratory analyses in the MLR (The ManyBabies Consortium, 2020, Table 6) revealed that the observed effect size depends on the inclusion criterion applied to the data. In addition, a follow-up project assessing test-retest reliability found reliable effects across test sessions only with more stringent criteria (Schreiner et al., 2022). In infant studies, it is common to only include participants who contribute data in 80% or even 100% of the presented trials. Based on the few papers where inclusion criteria were reported in the MAs, we can infer that studies on IDS preference follow this general pattern. In contrast, the MLR used a criterion of a minimum 12.5% of the trials in their main analyses (1 trial per condition over 16 trials). This very loose criterion was used because the MLR included planned analyses regarding data loss. We opted for 75% of the data (i.e. 7 of 8 trials per condition) as a more stringent exclusion criterion, which is closer to literature standards – when reported – and which substantially reduced the amount of data analysed.

Applying the more stringent inclusion criterion left 54 estimates and 952 participants in the MLR. The estimated average effect size in the MLR increased somewhat to SMD =0.42 [0.31, 0.53]. This average effect size was in fact numerically larger than that in the community-augmented MA by SMD =−0.09 [−0.25, 0.07] (p = 0.25), but the confidence interval was wide. Other patterns in the main analyses are largely preserved.

5.4.7 Exploratory sensitivity analysis: Restricting the age range when investigating interactions between the MA and MLR

There was a wider range of infant ages among the studies included in the community-augmented MA (see Figure 3A in the main manuscript). We therefore also investigated the extent to which the results for the model investigating interactions between source and moderator variables held when only including studies within the same age range as the MLR (i.e., only including studies with an average participant age ranging between 3 and 15 months). The MA included n=86 studies after restricting the age range. We fit the same interaction model as in section 3.2 of the main manuscript, including the two-way interactions between source (MA vs. MLR) and each of the three key moderators (infant age, test language, and method). Overall, the results were similar to the results using the full dataset (cf., Table 11 and Figure 9). There was a significant interaction between source and method (b = −0.36 [−0.67, −0.06]; p =0.02). We also found a marginal, non-significant interaction between source and infant age (b = −0.04 [−0.09, 0.00]; p =0.06); however, the magnitude of the coefficient estimate was virtually identical to the estimate in the model including the full MA dataset. As in the model with the full dataset, there was no interaction between source and test language (b = −0.19 [−0.58, 0.20]; p =0.27).

Moderator	Est	CI	p-value
Intercept	0.34	[0.24, 0.44]	<0.0001
Source (centered)	0.03	[-0.16, 0.23]	0.698
Age (months; centered)	0.02	[-0.00, 0.05]	0.055
Test Language (Native vs. Other)	0	[-0.20, 0.19]	0.987
Method (HPP vs. Other)	0.05	[-0.10, 0.21]	0.486
Source * Age	-0.04	[-0.09, 0.00]	0.061
Source * Test Language	-0.19	[-0.58, 0.20]	0.265
Source * Method	-0.36	[-0.67, -0.06]	0.021

Table 11: *Meta-regression estimates of the moderator interaction model when restricting the age range of the MA. Intercept: estimated mean SMD when averaging across all (centered) moderators. Age (in months) is mean-centered. Test Language (Native vs. Other) and Method (HPP vs. Other) are treated as a binary variables and centered. Bracketed values are 95% confidence intervals.*

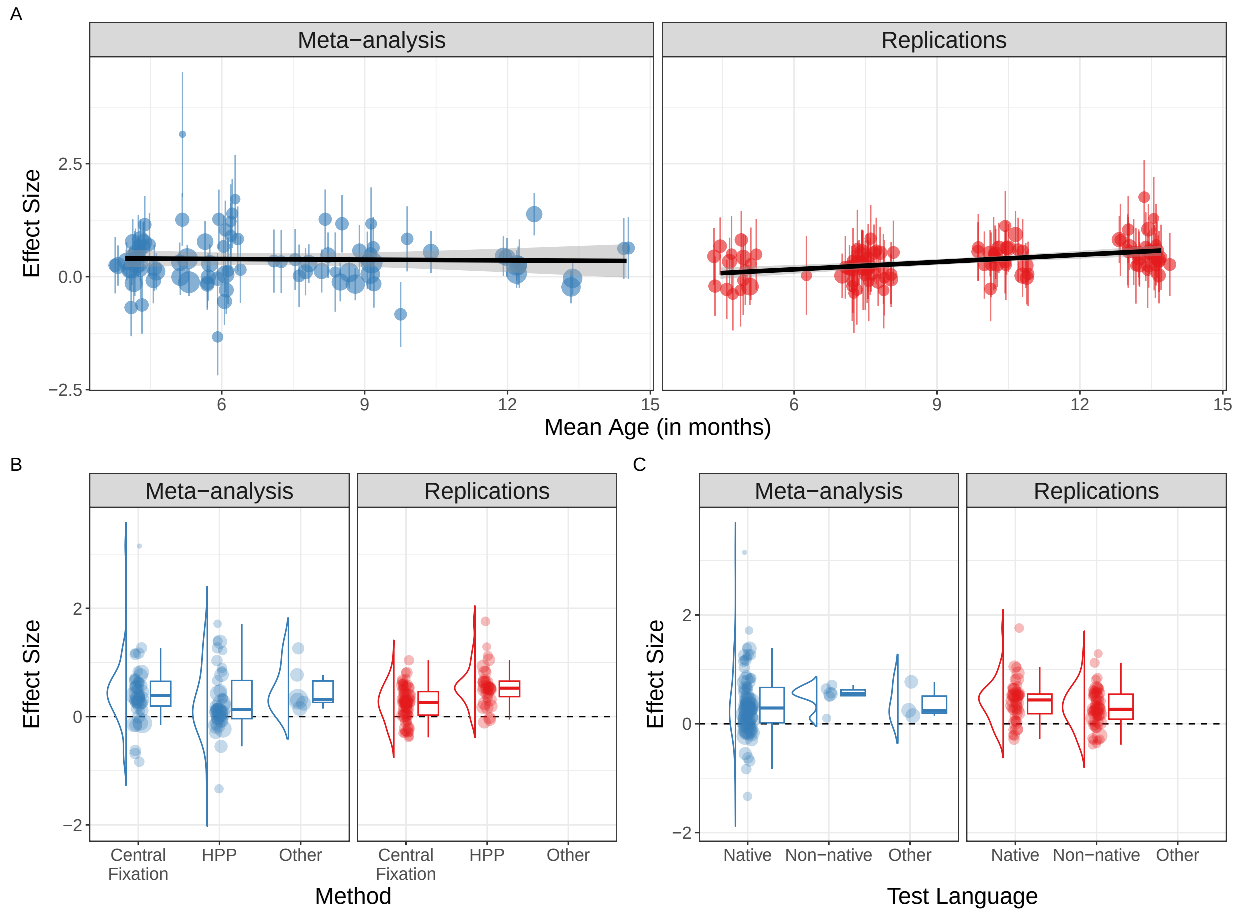

Figure 9: *Overview of the distribution of effect sizes in the meta-analytic dataset and the MLR restricted to studies with average ages between 3 and 15 months for three key moderators: age (A), method (B), and test language (C). In (A), the black line represents a linear fit through the effect sizes for each source and error bars for individual estimates are 95% confidence intervals.*

6. Discussion of discrepancies between results across MA datasets

In this section, we outline and contextualize the differences in results across the original, revised and community-augmented MA datasets (cf. Section 5.1 and 5.2 in Supplementary Materials to see the full results; a high-level overview of the key differences is provided in Section 5.3, Table 8). Specifically, we clarify how the goals of the study and our interpretations changed with each update to the dataset and explain why we believe that the community-augmented MA provides the most comprehensive meta-analytic estimate currently available.

When we first planned to compare the results from the MA to the MLR, we were faced with an intriguing puzzle: the original Dunst meta-analysis found an effect size almost twice as large (d = 0.67) as the effect size in the ManyBabies MLR (d = 0.35). On the surface, this discrepancy was also consistent with other findings suggesting that MAs tend to inflate effect sizes relative to MLRs (Kvarven et al., 2020). What could explain such a large discrepancy? One likely explanation was publication bias: MAs could be inflated in part because non-significant studies are more likely to be file-drawered than significant studies, artificially raising the estimates in the pool of published effects. However, past analyses suggested that publication bias could not fully explain the discrepancies observed between MAs and MLRs (Lewis et al., 2022), leading us to consider an alternative possibility: perhaps differences between MLRs and MAs could be explained due to systematic variation in key moderators. Consistent with this idea, the ManyBabies 1 project found that the magnitude of effect sizes varied across several key design (e.g., experiment method) and participant characteristics (e.g., infant age) within the MLR. We reasoned that accounting for the effect of moderators might reduce differences in the estimates of IDS preference between the MA and MLR. When we evaluated this possibility (cf., Section 5.1.), however, we found that in fact the opposite was true: accounting for the effect of key moderators substantially increased the discrepancy in the estimates for the MA vs. the MLR, from 0.29 to 0.45 (Table 2). Publication bias on its own also could not fully account for the discrepancy between the MA and the MLR. Initially, therefore, we were faced with an even more puzzling result than the unadjusted difference in estimates between the MA and the MLR — not only could we not account for this dramatic difference by testing what appeared to be the most promising explanations, our attempts to account for sources of variation between the MA and MLR in fact made the difference more pronounced.

Part of the solution to this puzzle appears to be simply error in the original MA. In the course of re-coding moderator variables for the original MA, we discovered a series of decisions and errors that required revision. These issues ranged from including duplicated effect sizes, incompatibility between the effect sizes reported in the MA and data reported in the paper, and the omission of several experiments with non-significant findings from papers otherwise included in the meta-analysis (cf. Section 2 in Supplementary Materials). Revising the meta-analysis in light of these issues substantially lowered the overall effect size estimate (to 0.50). Using the revised meta-analysis, we still found that accounting for moderators did not reduce discrepancies between the MA and MLR (though we also no longer found that accounting for moderators significantly increased discrepancies). On the other hand, we found that — across several methods — accounting for publication bias could in fact

explain the remaining discrepancy between the MA and the MLR. Under the assumption of "typical" publication bias in the field, the MA effect size estimate was numerically smaller than the MLR. Given the documented inconsistencies in the original Dunst meta-analysis, we are thus inclined to treat the puzzles raised by the comparison of the MLR to the original Dunst meta-analysis as entirely an artifact of errors in the original MA. Once these errors were revised, the meta-analytic estimate moved substantially closer to the MLR estimate and the puzzling increase in MA-MLR differences when accounting for moderator variables was no longer as marked.

While recoding and revising the Dunst et al. meta-analysis, the metalab community also worked on substantially augmenting the meta-analysis, leading to a dataset that included almost twice the number of studies in the revised meta-analysis. Comparing the MLR to this community-augmented MA clarified the picture dramatically: the updated meta-analytic estimate and the MLR estimate were almost completely aligned. By combining data from both a large-scale MA and MLR, we could therefore provide a more comprehensive picture of the generalizability of IDS preference, as reported in the main manuscript. At the same time, comparing these sources raised a new puzzle: unlike in the previous analyses, we now observed significant interactions between two key moderators (infant age and experiment method) and the data source. Both the Dunst et al. meta-analysis and the MLR originally reported a similar positive relationship between age and effect size magnitude, such that IDS preference increased with age in models combining the two data sources. In the community-augmented MA, we no longer observed an age effect, and any effect of method went, if anything, in the opposite direction from the increased effect size for the HPP method observed in the MLR.

Overall, we believe that the community-augmented MA provides the most accurate, comprehensive meta-analytic estimate currently available. The fact that its estimate converges with the effect size observed in the MLR increases confidence in the robustness and precision of our current "best estimate" of IDS preference of $d \approx 0.35$. At the same time, the differences in the moderating effects observed within each data source highlight the limitations of both the MA and the MLR to fully address questions of key theoretical and methodological interest, such as the developmental trajectory of IDS preference and its dependence on different methods for eliciting preference. We hope that these remaining puzzles can motivate future work in the field and catalyze improvements in how we approach knowledge building from both MAs and MLRs. Here we highlight the value of manipulating key variables of interest to discover how effects, such as IDS preference, vary across a broad terrain of theoretically relevant dimensions.

References

Barker, B. A., & Newman, R. S. (2004). Listen to your mother! the role of talker familiarity in infant streaming. *Cognition*, *94*(2), B45–B53.

Bergmann, C., Tsuji, S., Piccinini, P. E., Lewis, M. L., Braginsky, M., Frank, M. C., & Cristia, A. (2018). Promoting replicability in developmental research through meta-analyses: Insights from language acquisition research. *Child Development*, *89*(6), 1996–2009.

Byers-Heinlein, K., Tsui, A. S. M., Bergmann, C., Black, A. K., Brown, A., Carbajal, M. J., ... Wermelinger, S. (2021). A multilab study of bilingual infants: Exploring the preference for infant-directed speech. *Advances in Methods and Practices in Psychological Science*, *4*(1), 1–30. doi: 10.1177/2515245920974622

Cooper, R. P., & Aslin, R. N. (1990). Preference for infant-directed speech in the first month after birth. *Child Development*, *61*, 1584–1595.

Cox, C., Bergmann, C., Fowler, E., Keren-Portnoy, T., Roepstorff, A., Bryant, G., & Fusaroli, R. (2022). A systematic review and bayesian meta-analysis of the acoustic features of infant-directed speech. *Nature Human Behaviour*, *7*, 114-133. doi: 10.1038/s41562-022-01452-1

Dunst, C., Gorman, E., & Hamby, D. (2012). Preference for infant-directed speech in preverbal young children. *Center for Early Literacy Learning*, *5*(1), 1–13.

Fernald, A. (1985). Four-month-old infants prefer to listen to motherese. *Infant Behavior and Development*, *8*(2), 181–195. Retrieved 2023-05-31, from https://linkinghub.elsevier.com/retrieve/pii/S0163638385800059 doi: 10.1016/S0163-6383(85)80005-9

Fernald, A., Taeschner, T., Dunn, J., Papousek, M., de Boysson-Bardies, B., & Fukui, I. (1989). A cross-language study of prosodic modifications in mothers' and fathers' speech to preverbal infants. *Journal of child language*, *16*(3), 477–501.

Hedges, L. V., Tipton, E., & Johnson, M. C. (2010). Robust variance estimation in meta-regression with dependent effect size estimates. *Research Synthesis Methods*, *1*(1), 39–65.

Kvarven, A., Strømland, E., & Johannesson, M. (2020). Comparing meta-analyses and preregistered multiple-laboratory replication projects. *Nature Human Behaviour*, *4*(4), 423–434. doi: https://doi.org/10.1038/s41562-019-0787-z

Lewis, M., Mathur, M., VanderWeele, T., & Frank, M. C. (2022). The puzzling relationship between multi-lab replications and meta-analyses of the rest of the literature. *Royal Society Open Science*, *9*(2), 211499.

Mathur, M. B., & VanderWeele, T. J. (2020a). Robust metrics and sensitivity analyses for meta-analyses of heterogeneous effects. *Epidemiology*, *31*(3), 356–358.

Mathur, M. B., & VanderWeele, T. J. (2020b). Sensitivity analysis for publication bias in meta-analyses. *Journal of the Royal Statistical Society: Series C*, *5*(69), 1091–1119.

Mathur, M. B., & VanderWeele, T. J. (2021). Meta-regression methods to characterize evidence strength using meaningful-effect percentages conditional on study characteristics. *Research Synthesis Methods*, *12*(6), 731-749.

Schachner, A., & Hannon, E. E. (2011). Infant-Directed Speech Drives Social Preferences in 5-Month-Old Infants. *Developmental Psychology*, *47*(1), 19–25. doi: 10.1037/a0020740

Schreiner, M. S., Zettersten, M., Bergmann, C., Frank, M. C., Fritzsche, T., Gonzalez-Gomez, N., ... Lippold, M. (2022, December). *Limited evidence of test-retest reliability in infant-directed speech preference in a large pre-registered infant sample.* PsyArXiv. Retrieved 2023-06-06, from `https://psyarxiv.com/uwche/` doi: 10.31234/osf.io/uwche

The ManyBabies Consortium. (2020). Quantifying sources of variability in infancy research using the infant-directed-speech preference. *Advances in Methods and Practices in Psychological Science*, *3*(1), 24–52. doi: 10.1177/2515245919900809

Tipton, E., Pustejovsky, J. E., & Ahmadi, H. (2019). A history of meta-regression: Technical, conceptual, and practical developments between 1974 and 2018. *Research synthesis methods*, *10*(2), 161-179.

van Rooijen, R., Bekkers, E., & Junge, C. (2019). Beneficial effects of the mother's voice on infants' novel word learning. *Infancy*, *24*(6), 838–856.

Vevea, J. L., & Hedges, L. V. (1995). A general linear model for estimating effect size in the presence of publication bias. *Psychometrika*, *60*(3), 419–435. (https://doi.org/10.1007/BF02294384)
